# Spectral Analysis of Input Spike Trains by Spike-Timing-Dependent Plasticity

**DOI:** 10.1371/journal.pcbi.1002584

**Published:** 2012-07-05

**Authors:** Matthieu Gilson, Tomoki Fukai, Anthony N. Burkitt

**Affiliations:** 1 Riken Brain Science Institute, Wako-shi, Saitama, Japan; 2 NeuroEngineering Laboratory, Department of Electrical and Electronic Engineering, The University of Melbourne, Melbourne, Victoria, Australia; 3 Centre for Neural Engineering, The University of Melbourne, Melbourne, Victoria, Australia; 4 Bionics Institute, East Melbourne, Victoria, Australia; Indiana University, United States of America

## Abstract

Spike-timing-dependent plasticity (STDP) has been observed in many brain areas such as sensory cortices, where it is hypothesized to structure synaptic connections between neurons. Previous studies have demonstrated how STDP can capture spiking information at short timescales using specific input configurations, such as coincident spiking, spike patterns and oscillatory spike trains. However, the corresponding computation in the case of arbitrary input signals is still unclear. This paper provides an overarching picture of the algorithm inherent to STDP, tying together many previous results for commonly used models of pairwise STDP. For a single neuron with plastic excitatory synapses, we show how STDP performs a spectral analysis on the temporal cross-correlograms between its afferent spike trains. The postsynaptic responses and STDP learning window determine kernel functions that specify how the neuron “sees” the input correlations. We thus denote this unsupervised learning scheme as ‘kernel spectral component analysis’ (kSCA). In particular, the whole input correlation structure must be considered since all plastic synapses compete with each other. We find that kSCA is enhanced when weight-dependent STDP induces gradual synaptic competition. For a spiking neuron with a “linear” response and pairwise STDP *alone*, we find that kSCA resembles principal component analysis (PCA). However, plain STDP does not isolate correlation sources in general, e.g., when they are mixed among the input spike trains. In other words, it does not perform independent component analysis (ICA). Tuning the neuron to a single correlation source can be achieved when STDP is paired with a homeostatic mechanism that reinforces the competition between synaptic inputs. Our results suggest that neuronal networks equipped with STDP can process signals encoded in the transient spiking activity at the timescales of tens of milliseconds for usual STDP.

## Author Summary

Tuning feature extraction of sensory stimuli is an important function for synaptic plasticity models. A widely studied example is the development of orientation preference in the primary visual cortex, which can emerge using moving bars in the visual field. A crucial point is the decomposition of stimuli into basic information tokens, e.g., selecting individual bars even though they are presented in overlapping pairs (vertical and horizontal). Among classical unsupervised learning models, independent component analysis (ICA) is capable of isolating basic tokens, whereas principal component analysis (PCA) cannot. This paper focuses on spike-timing-dependent plasticity (STDP), whose functional implications for neural information processing have been intensively studied both theoretically and experimentally in the last decade. Following recent studies demonstrating that STDP can perform ICA for specific cases, we show how STDP relates to PCA or ICA, and in particular explains the conditions under which it switches between them. Here information at the neuronal level is assumed to be encoded in temporal cross-correlograms of spike trains. We find that a linear spiking neuron equipped with pairwise STDP requires additional mechanisms, such as a homeostatic regulation of its output firing, in order to separate mixed correlation sources and thus perform ICA.

## Introduction

Organization in neuronal networks is hypothesized to rely to a large extent on synaptic plasticity based on their spiking activity. The importance of spike timing for synaptic plasticity has been observed in many brain areas for many types of neurons [1], [2], which was termed spike-timing-dependent plasticity (STDP). On the modeling side, STDP was initially proposed to capture information within spike trains at short timescales, as can be found in the auditory pathway of barn owls [3]. For more than a decade, STDP has been the subject of many theoretical studies to understand how it can select synapses based on the properties of pre- and postsynaptic spike trains. A number of studies have focused on how STDP can perform input selectivity by favoring input pools with higher firing rates [4], [5], with synchronously firing inputs [6], or both [7], detect spike patterns [8] and rate-modulated patterns [9], and interact with oscillatory signals [10], [11]. The STDP dynamics can simultaneously generate stability of the output firing rate and competition between individual synaptic weights [6], [12]–[15]. In order to strongly drive the postsynaptic neurons, which we refer to as robust neuronal specialization. [16]. When considering recurrently connected neurons, the weight dynamics can lead to emerging functional pathways [17]–[19] and specific spiking activity [20], [21]. Recent reviews provide an overview of the richness of STDP-based learning dynamics [22], [23].

The present paper aims to provide a general interpretation of the synaptic dynamics at a functional level. In this way, we want to characterize how spiking information is relevant to plasticity. Previous publications [24], [25] mentioned the possible relation between STDP and Oja's rate-based plasticity rule [26], which performs principal component analysis (PCA). Previous work [27] showed how STDP can capture slow time-varying information within spike trains in a PCA-like manner, but this approach does not actually make use of the temporal (approximate) antisymmetry of the typical STDP learning window for excitatory synapses; see also earlier work about storing correlations of neuronal firing rates [28]. Along similar lines, STDP was used to perform independent component analysis (ICA) for specific input signals typically used to discriminate between PCA and ICA [7], [29]. In particular, STDP *alone* did not seem capable of performing ICA in those numerical studies: additional mechanisms such as synaptic scaling were necessary. On the other hand, additive-like STDP has been shown to be capable of selecting only one among two identical input pools with independent correlations from each other, also referred to as ‘symmetry breaking’ [13], [17]. In addition to studies of the synaptic dynamics, considerations on memory and synaptic management (e.g., how potentiated weights are maintained) have been used to relate STDP and optimality in unsupervised learning [30], [31]. To complement these efforts, the present paper proposes an in-depth study of the learning dynamics and examines under which conditions pairwise STDP can perform ICA. For this purpose, we consider input spiking activity that mixes correlation sources. We draw on our previously developed framework that describes the weight dynamics [15], [23] and extend the analysis to the case of an *arbitrary* input correlation structure. This theory is based on the Poisson neuron model [6] and focuses on pairwise weight-dependent STDP for excitatory synapses. Mutual information is used to evaluate how STDP modifies the neuronal response to correlated inputs [32]. This allows us to relate the outcome of STDP to either PCA and ICA [33]. Finally, we examine the influence of the STDP and neuronal parameters on the learning process. Our model captures fundamental properties shared by more elaborate neuronal and STDP models. In this way, it provides a minimal and tractable configuration to study the computational power of STDP, bridging the gap between physiological modeling and machine learning.

## Results

**Figure 1:**
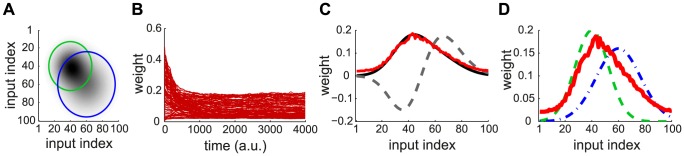
Example of PCA performed by Oja's rate-based plasticity rule. (A) Theoretical cross-correlation matrix of 100 inputs induced by two Gaussian distributions of rates. Darker pixels indicate higher correlations. The circles indicate where the correlation for each source drops below 10% of its maximum (see the Gaussian profiles in D). (B) Traces of the weights modified by Oja's rule [26] in (1). At random times, the input rates 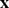 follow either Gaussian rate profile corresponding to the green and blue curves in D; white noise is added at each timestep. (C) The asymptotic distribution of the weights (red) is close to the principal component of the matrix in A (black solid curve), but distinct from the second component (black dashed curve). (D) The final weight distribution (red) actually overlaps both Gaussian rate profiles in green dashed and blue dashed-dotted lines that induce correlation in the inputs. The green and blue curves correspond to the small and large circles in A, respectively.

Spectral decomposition is typically used to find the meaningful components or main trends in a collection of input signals (or data). In this way, one can represent or describe the inputs in a summarized manner, i.e., in a space of lower dimension. This paper focuses on the information conveyed by spike trains, which will be formalized later. The function of neuronal processing is to extract the dominant component(s) of the information that it receives, and disregard the rest, such as noise. In the context of learning, synaptic competition favors some weights at the expense of others, which tunes the neuronal selectivity. As a first step to introduce spectral decomposition, we consider Oja's rule [26] that enables a linear non-spiking neuron to learn the correlations between its input firing rates. At each time step, the 100 input firing rates are determined by two Gaussian profiles with distinct means, variances and amplitudes (green and blue curves in Fig. 1D), in addition to noise. The area under the curve indicates the strength of input correlations; here the green dashed curve “dominates” the blue dashed-dotted curve. This results in correlation among the input rates, as represented by the matrix in Fig. 1A. The vector of weights 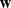 is modified by Oja's rule:
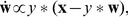
(1)where 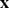 is the input rates and 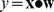 is the neuron output (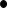 indicates the scalar product of the two vectors). The weight evolution is represented in Fig. 1B. The final weight distribution reflects the principal component of the correlation matrix (red solid curve in Fig. 1C). As shown in Fig. 1D, this does not represent only the stronger correlation source (green dashed curve), but also the weaker one (blue dashed-dotted curve). This follows because the principal component mixes the two sources, which overlap in Fig. 1A. In other words, Oja's rule cannot isolate the strongest source and thus cannot perform ICA, but only PCA. We will examine later whether the same phenomenon occurs for STDP. Note that the rate correlation matrix is always symmetric. This differs from using PCA in the context of data analysis, such as finding the direction that provides the dependence of highest magnitude in a cloud of data points.

### Spiking neuron configuration

**Figure 2:**
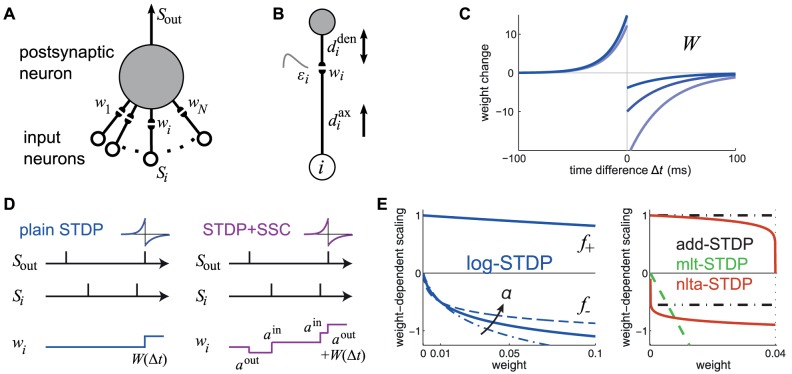
Single neuron with STDP-plastic excitatory synapses. (A) Schematic representation of the neuron (top gray-filled circle) and the 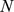 synapses (pairs of black-filled semicircles) that are stimulated by the input spike trains 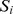 (bottom arrows). (B) Detail of synapse 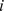, whose weight is 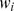, postsynaptic response kernel 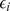, axonal and dendritic delays 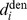 and 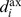, respectively. The arrows indicate that 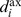 describes the propagation along the axon to the synapse, while 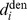 relates to both conduction of postsynaptic potential (PSP) toward soma and back-propagation of action potential toward the synaptic site. (C) Example of temporally Hebbian weight-dependent learning window 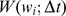 that determines the STDP contribution of pairs of pre- and postsynaptic spikes. The curve corresponds to (22). Darker blue indicates a stronger value for 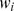, which leads to less potentiation and more depression. (D) Schematic evolution of the weight 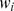 for given pre- and postsynaptic spike trains 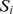 and 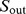. The size of each jump is indicated by the nearby expression. Comparison between plain STDP for which only pairs contribute and STDP+SCC where single spikes also modify the weight via the terms 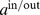. Here only the pair of latest spikes falls into the temporal range of STDP and thus significantly contributes to STDP. (E) Scaling functions of 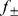 that determine the weight dependence for LTP and LTD. In the left panel, the blue solid curve corresponds to log-STDP [16] with 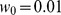, 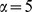 and 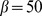 in (23). The parameter 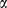 controls the saturation of the LTD curve: the dashed curve corresponds to 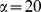 and the dashed-dotted curve to 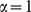. In the right panel, the red solid curves represent 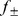 for nlta-STDP [13] with 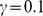 and 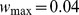 in (24); the black dashed-dotted horizontal lines indicate the add-STDP that is weight independent; the green dashed line corresponds to a linearly dependent LTD for mlt-STDP [38].

In order to examine the computational capabilities of STDP, we consider a single neuron whose 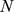 excitatory synapses are modified by STDP, as shown in Fig. 2A. Our theory relies on the Poisson neuron model, which fires spikes depending on a stochastic rate intensity that relates to the soma potential. Each presynaptic spike induces variation of the soma potential, or postsynaptic potential (PSP), described by the normalized kernel function 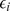, shifted by the axonal and dendritic delays, 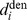 and 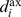, respectively (Fig. 2B). The size of the PSP is scaled by the synaptic weight 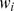.

#### Pairwise weight-dependent STDP model

We use a phenomenological STDP model described by a learning window 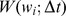 as in Fig. 2C. Importantly, LTP/LTD is not determined by the relative timing of firing at the neuron somas, but by the time difference at the synaptic site, meaning that 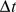 incorporates the axonal and dendritic delays. This choice can be related to more elaborate plasticity models based on the local postsynaptic voltage on the dendrite [7], [34].

We will examine common trends and particularities of the weight specialization for several models of STDP.

- A “plain” STDP model postulates that all pairs of pre- and postsynaptic spikes, and only them, contribute to the weight modification, provided the time difference 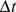 is in the range of the learning window 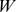 as illustrated in Fig. 2D.
- A second scheme assumes that, in addition to STDP-specific weight updates, each pre- or postsynaptic spike also induces a weight update via the corresponding contribution 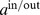, as illustrated in Fig. 2D. This will be referred to as ‘STDP+SSC’, as opposed to ‘plain STDP’ (or ‘STDP’ alone when no precision is needed). Although sometimes regarded as less plausible from a biological point of view, single-spike contributions can regulate the neuronal output firing in a homeostatic fashion [35], [36]. In particular, we will examine the role of 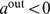 that has been used to enhance the competition between synaptic inputs [6].
- We will consider weight dependence for STDP, namely how the learning window function 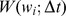 depends on the weight 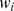 as in Fig. 2C, following experimental observations [37]. Figure 2E represents four examples of weight dependence: our ‘log-STDP’ in blue [16], the weight-*independent* ‘add-STDP’ for additive STDP [6], [12], ‘nlta-STDP’ proposed by Gütig et al. [13], and ‘mlt-STDP’ for the multiplicative STDP by van Rossum et al. [38] in which LTD scales linearly with 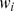. For log-STDP and nlta-STDP, the weight dependence can be adjusted via a parameter. For log-STDP (left panel), the LTD curve scales almost linearly with respect to 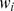 for small values of 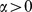 in a similar manner to mlt-STDP, whereas it is additive-like (weight-independent) STDP for large values of 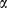 (in the range 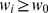). Likewise, nlta-STDP scales between the other ‘multiplicative’ STDP proposed by Rubin et al. [39] for 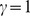 and add-STDP for 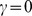; the red curve in Fig. 2E uses 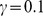.

Variability is also incorporated in the weight updates through the white noise 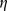, although its effect will not be examined specifically in the present work. Typical parameters used in simulations are given in Table 1 and detailed expressions for 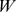 are provided in Methods.

**Table 1:** Neuronal and learning parameters.

Quantity:	variable name and value
time step	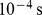
simulation duration	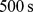
**Input parameters**	
input firing rate	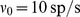
input correlation strength	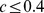
**PSP parameters**	
synaptic rise time constant	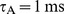
synaptic decay time constant	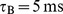
axonal delays	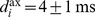
dendritic delays	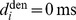
**STDP model**	
learning speed	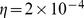
LTP time constant	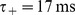
LTD time constant	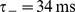
white noise standard deviation	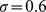
**log-STDP in (23)**	
LTP scaling coefficient	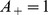
LTP decay factor	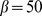
LTD scaling coefficient	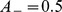
LTD curvature factor	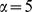
reference weight	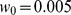
**nlta-STDP in (24)**	
LTP scaling coefficient	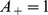
LTD scaling coefficient	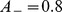
weight-dependence exponent	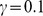
weight upper bound	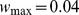
**add-STDP**	
LTP scaling coefficient	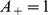
LTD scaling coefficient	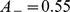
weight upper bound	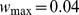
**mlt-STDP in (25)**	
LTP scaling coefficient	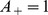
LTD scaling coefficient	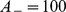
**single-spike plasticity terms (SCC)**	
presynaptic contribution	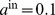
postsynaptic contribution	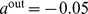

Unless specified, the above parameters are used in numerical simulation.

#### Learning dynamics

**Figure 3:**
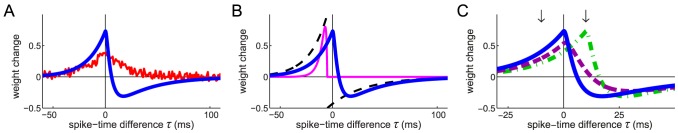
Kernel function 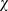. (A) Anticonvolution of a fictive correlogram 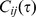 (red curve) and a typical kernel function 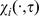 (blue curve). The amount of LTP/LTD corresponds to the area under the curve of the product of the two functions. (B) Plot of a typical kernel 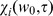 as a function of 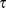 (blue curve). It corresponds to (4) for log-STDP with the baseline parameters in Table 1, namely rise and decay time constants 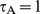 and 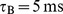 in (29), respectively, and a purely axonal delay 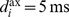. The related STDP learning window 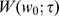 is plotted in black dashed line and the mirrored PSP response in pink solid line. The effect of the axonal delay shifts both the 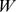 and the PSP in the same direction, which cancels out. (C) Variants of 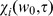 for longer PSP time constants, 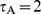 and 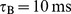 (purple curve); and for a dendritic delay 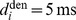 (green dashed-dotted curve). In contrast to 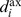 that does not play a role in (4), 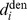 shifts 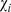 to the right. The arrows indicate 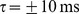.

The present analysis is valid for any pairwise STDP model that is sensitive to up-to-second order spike-time correlations. In its present form, it cannot deal with, for example, the ‘triplet’ STDP model [40] and the stochastic model proposed by Appleby and Elliott [41]. The neuronal spiking activity is described by the corresponding firing rates and spike-time correlations. See Table 2 for an overview of the variables in our system. The input rates and correlations are assumed to be consistent over the learning epoch. Details of the analytical calculations are provided in Methods. The evolution of the vector of plastic weights 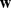 is then governed by the following differential equation:
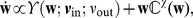
(2)where the dependence over time 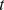 is omitted. The function 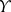 lumps rate contributions to plasticity (including STDP) and depends on the vector of input firing rates 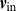 and neuronal output firing rate 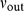, as well as the weights. The second term describes STDP-specific spike-based effects. The STDP effect are described by the matrix 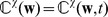, which is assumed to be independent of 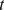 and whose elements are:
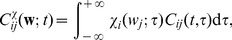
(3)namely the (anti)convolution of the input spike-time cross-correlograms 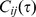 with the kernel functions 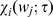, for each pair of inputs 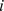 and 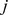. A schematic example is illustrated in Fig. 3A. For clarity purpose, we rewrite the time difference 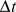 as 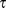 hereafter. In (3), each kernel 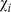 combines the STDP learning window at synapse 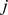 and the postsynaptic response kernels 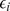:
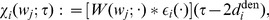
(4)where the convolution indicated by 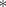 concerns the variable 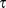, as illustrated in Fig. 3B. For weight-dependent STDP, the kernel is modified via the scaling of both potentiation and depression for 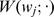 in terms of 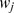 (Fig. 2C). In addition, the postsynaptic response crucially shapes 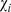 [19], [27], as shown in Fig. 3C. In the case of a single neuron (as opposed to a recurrent network), the dendritic delay 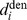 plays a distinct role compared to the axonal delay 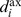 in that it shifts the kernel 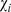 as a function of 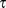 to the right, namely implying more potentiation for 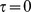.

**Table 2:** Variables and parameters that describe the neuronal learning system.

Description	symbol	(vector/matrix notation)
input firing rates	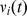	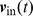
input spike-time cross-covariances	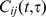	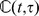
neuronal firing rate	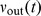	
input-output spike-time covariances	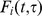	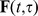
synaptic weights	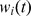	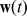
PSP function	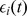	
axonal delays	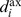	
dendritic delays	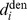	
kernel functions for synapse 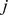	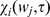	
lumped plasticity rate-based effects		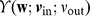
STDP-specific plasticity spike effects	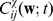	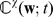
integral value of STDP	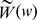	

The variable 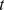 denotes the time, whereas 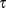 indicates the spike-time difference (or time lag) used in correlations and covariances.

### Encoding the input correlation structure into the weight structure

We stress that the novel contribution of the present work lies in considering general input structures, i.e., when the matrix of cross-correlograms 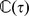 is arbitrary. This extends our previous study [15] of the case of homogeneous within-pool correlations and no between-pool correlations, the matrix 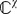 in (3) is diagonal (by block). We focus on the situation where the average firing rates across inputs do not vary significantly. This means that rate-based plasticity rules cannot extract the spiking information conveyed by these spike trains. In this case, pairwise spike-time correlations mainly determine the weight specialization induced by STDP via 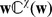, dominating rate effects lumped by 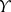 in (2). The key is the spectral properties of 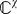, which will be analyzed as follows:

- evaluation of the equilibrium value for the mean weight 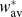 in the uncorrelated case;
- calculation of the matrix 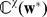 that combines the input correlation structure, the PSP and STDP parameters (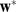 is the homogeneous weight vector for which 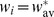 for all 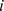);
- analysis of the spectrum of 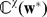 to find the dominant eigenvalue(s) and the corresponding left-eigenvector(s);
- decomposition of the initial weight structure (e.g., homogeneous distribution) in the eigenspace to predict the specialization.

#### Equilibrium for the mean weight 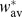


Partial stability of the synaptic dynamics is necessary in order that not all weights cluster at zero or tend to continuously grow. This also implies the stabilization of the output firing rate. Here we require that the STDP dynamics itself provides a stable fixed point for 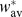. In particular, this must be true for uncorrelated inputs, which relates to equating to zero the term 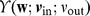 in (2).

For plain STDP with weight dependence, the corresponding fixed point is determined by the STDP learning window alone, which is the same for all weights 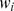 here and is related to the integral value
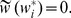
(5)Here the weight dependence alone can stabilize the mean synaptic weights, which requires that 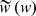 decreases when 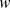 increases [13], [38], [42].

For STDP+SCC, a mean-field approximation of 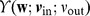 over the pool of incoming synapses is often used to evaluate the stability of the mean weight 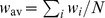, which gives 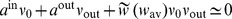, where 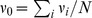 is the mean input firing rate. The equilibrium values for the mean weight 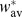 and the neuronal firing rate 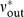 are then related by
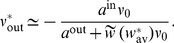
(6)A stable fixed point for arbitrary input configuration is ensured by 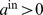, 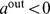 and a negative derivative 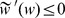 as a function of 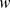 as well as 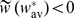 at the weight equilibrium value [19]. This means that the right-hand side is a negative function of 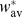. Note that the additional condition 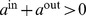 is required for networks with plastic recurrent synapses [43]. The plasticity terms 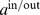 can lead to a homeostatic constraint on the output firing rate 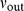 [35]. In the case of stability, the equilibrium values 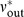 and 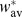 depend on the respective input firing rate 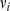. For weight-dependent STDP+SCC, fixed points 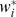 also exist for individual weights 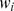 and correspond to (6) when replacing 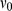 by 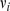 and 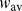 by 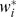. The implications of these two different ways of stabilizing 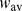 will be discussed via numerical results later.

#### Spectrum of 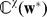 and initial weight specialization

Following our previous study [16], we consider that rate-based effects vanish and focus on the initial stage when weights specialize due to the spike-time correlation term involving 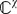 in (2). This means that we approximate
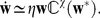
(7)The weight evolution can be evaluated using (7) provided spike-time correlations are significantly strong compared to the “noise” in the learning dynamics. The rate terms in 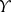 are proportional to the 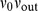 whereas the spike-based term grow with 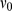 only. This implies stronger noise and more difficulty to potentiate weights when 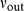 is high at the baseline state, e.g., for large input firing rates. Assuming homogeneous weights 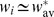 as initial condition, the weight dynamics is determined by the learning window 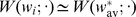. As a first step, we consider the case where the matrix 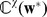 is diagonalizable as a real matrix, namely 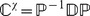 with 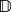 a diagonal matrix and 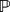 the matrix for the change of basis (all with real elements). The rows of 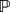 are the orthogonal left-eigenvectors 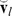 corresponding to the eigenvalues 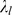, 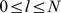 that are the diagonal elements of 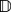. The weight vector 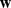 can be decomposed in the basis of eigenvectors (or spectral components)
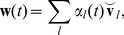
(8)where 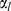 are the coordinates of 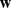 in the new basis. By convention, we require all 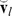 to be normalized and that 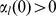 at time 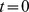 for 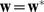. Transposing (7) in the new basis, the evolution of 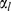 can be approximated by 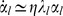, which gives
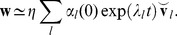
(9)The initial weight specialization is thus dominated by the 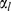 related to the largest positive eigenvalues 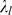 and can be predicted by the corresponding eigenvectors 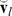 [19], [25].

In general, we can use the property that the set of diagonalizable matrices with complex elements is dense in the vector space of square matrices [44, p 87]. This means that it is possible to approximate 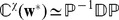, in which case 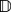 and 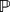 may have non-real elements. If the eigenvalue with the largest real part is a real number, the same conclusion as above is expected to hold, even though the eigenvectors may not be orthogonal. When a pair of eigenvalues dominate the spectrum, 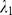 and its conjugate 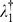. The decomposition of the homogeneous vector 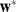 on the plane of the corresponding eigenvectors that gives 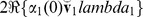 leads to the dominant term 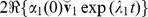 in the equivalent to (9); 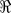 denotes the real part here. The initial growth or decay of 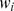 is given by the derivative:
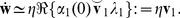
(10)Note that this expression applies to the real case too, where 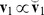 and the convention 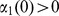 simply means that 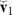 reflects the signs of the elements of the derivative vector.

In most cases, the spectrum is dominated as described above and we can use the expression (10), which will be referred to as the ‘strongest’ spectral component of 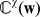. Note that, in the case of a non diagonalizable matrix, the Jordan form of 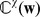 could be used to describe more precisely the weight evolution, for example. We have also neglected the case 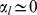 for 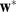, for which the decomposition of the before-learning weight specialization 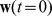 may also play a role. Nevertheless, noise in the weight dynamics will lead the system away from such unstable fixed points.

#### Asymptotic weight structure and stability

**Figure 4:**
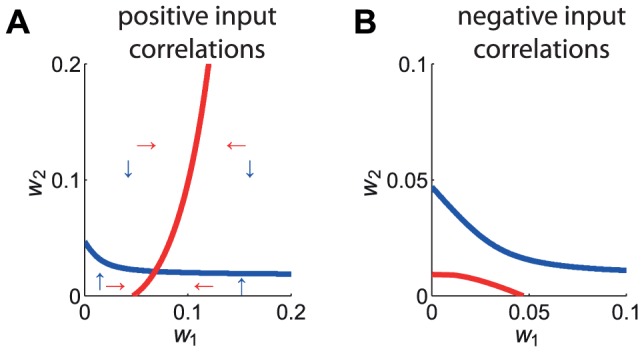
Existence of a fixed point for the weight dynamics. (A) Curves of the zeros of (39) for 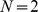 weights in the case of positively correlated inputs. The two curves have an intersection point, as the equilibrium curve for 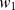 in red spans all 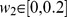, while that for 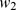 in blue spans all 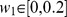. The arrows indicate the signs of the derivatives 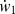 and 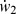 in each quadrant (red and blue, resp.). (B) Similar to A with negative input correlations, for which the curves do not intersect.

Now we focus on the final weight distribution that emerges, following the initial splitting. In particular, a stable asymptotic structure can be obtained when the learning equation (2) has a (stable) fixed point, as illustrated in Fig. 4A for the simple case of two weights 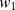 and 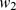. Weight dependence can lead to the existence of at least a realizable and stable fixed point. Two conditions ensure the existence of a solution to the learning equation. First, the weight dependence should be such that LTD vanishes for small weights while LTP vanishes for large weights, as is the case for both log-STDP and nlta-STDP for 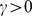. Second, the inputs should be positively correlated. If this second assumption is lifted, the fixed point may become unrealizable (e.g., 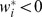) or simply not exist as in Fig. 4B. Nevertheless, we can also conclude the existence of a stable fixed point in the range of *small* negative correlations. This follows because of the continuity of the matrix coefficients in (2), which determines the fixed points 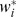, with respect to the matrix elements of 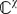.

A general result about the relationship between the fixed point(s) and 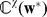 is a difficult problem because 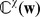 changes together with 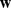 for a weight-dependent learning window 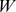. This implies that the eigenvector basis 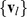 are modified together with 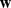. With the further assumption of a weak weight dependence and for a single dominant eigenvector 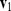, the term 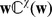, which determines the weight specialization, remains similar to 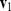. By this, we mean that the elements of both vectors are sorted in the same order. At the equilibrium, rate-based effects lumped in 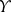 balance the spike-based effects that are qualitatively described by 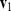. Under our assumptions, the vector elements of 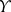 are decreasing functions of the weights 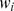. It follows that inputs corresponding to larger elements of (10) end up at a higher level of potentiation. However, when 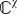 has a strong antisymmetric component due to negative matrix elements, it can exhibit complex conjugate dominant eigenvalues with large imaginary parts. The weight vector 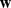 may experience a rotation-like evolution, in which case the final distribution differs qualitatively from the initial splitting. Nevertheless, the weights with strongest initial LTP are expected to be mostly potentiated eventually. Further details are provided in Methods. Deviation from the predictions can also occur when several eigenvalues with similar real parts dominate the spectrum.

In the particular case of *additive* STDP, a specific issue arises since the existence of a fixed point is not guaranteed. When 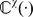 has purely complex eigenvalues, the weight dynamics are expected to exhibit an oscillatory behavior that may impair the emergence of an asymptotic weight structure, as was pointed out by Sprekeler et al. [27]. An example with add-STDP+SCC and eigenvalues that have large imaginary parts is provided in Text S1.

As some weights grow larger, they compete to drive the neuronal output firing [12]. This phenomenon is relatively weak for Poisson neurons compared to integrate-and-fire neurons [16]. For STDP+SCC, synaptic competition is enhanced when using 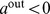. Following (6), the larger negative 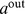 is, the lower the output firing rate 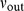 that is maintained by STDP at the equilibrium. This also holds when inputs are correlated. These rate effects lead to a form of sparse coding in which fewer weights are significantly potentiated, while the remainder weights are kept small. Another interpretation of the effect of 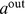 relies on the fact that all weights are homogeneously depressed after postsynaptic spiking. Then, only the weights of inputs involved in triggering firing may not experience depression provided STDP sufficiently potentiate them. This concerns inputs related to a common correlation source and may result in a winner-take-all situation. Moreover, this effect increases with the output firing rate and may become dominant when STDP generates strong LTP, leading to large weights.

In summary, for plain STDP, the final weight structure for plain STDP is expected to reflect the initial splitting, which is determined by the strong spectral component of 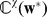 in (10), at least for the most potentiated weights that win the competition. The assumption of “sufficiently weak” weight dependence holds for log-STDP with 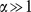 (sublinear saturation for 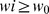) and for nlta-STDP with small values of 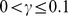 (for 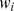 away from the bounds). STDP+SCC may modify the final weight distribution when the single-spike contributions have comparably strong effects to STDP. In particular, competition between correlation sources is expected to be enhanced when 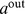 is sufficiently large negative. In the following sections, we verify these predictions using numerical simulation for various input configurations.

### Input spike-time correlation structure

In order to illustrate the above analysis, we consider input configurations that give to “rich” matrices of pairwise correlations 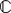. Model input spike trains commonly combine stereotypical activity and random “background” spikes. Namely, to predict the evolution of plastic synaptic weights, it is convenient that the statistical properties of the inputs are invariant throughout the learning epoch (e.g., the presentation of a single stimulus). Mathematically, we require the input spike trains to be second-order stationary. In this way, the input firing rates 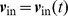 and the spike-time correlograms 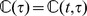 in (2) are well-defined and practically independent of time 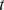, even though the spike trains themselves may depend on time. The formal definitions of 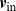 and 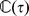 in Methods combine a stochastic ensemble average and a temporal average. This allows to deal with a broad class of inputs that have been used to investigate the effect of STDP, such as spike coordination [6], [13], [14], [36] and time-varying input signals that exhibit rate covariation [12], [27], [45], as well as elaborate configurations proposed recently [46]–[48]. Most numerical results in the present paper use the spike coordination that mixes input correlation sources. In the last section of Results, rate covariation will also be examined for the sake of generality.

#### Pools with mixed spike-time correlation

Inputs thus generated model signals that convey information via precise timing embedded in noisy spike trains. A simple example consists of instantaneously correlated spike trains that correspond to input neurons belonging to the same afferent pathway, which have been widely used to study STDP dynamics [13], [14], [36]. Here we also consider the situation where synapses can take part in conveying distinct independent signals, as well as time lags between the relative firing of inputs. To do so, spike trains are generated using a thinning of homogeneous Poisson processes. Namely, independent homogeneous Poisson processes are used as references 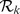 to determine correlated events at a given rate 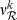 that trigger for some designated inputs. For input 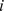, we denote 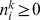 the number of spikes associated with each correlated event from 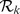. The probability of firing after a given latency 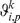 is 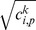 with 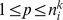. Outside correlated events, inputs randomly fire spikes such that they all have the same time-averaged firing rate 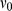. This corresponds to an additional Poisson process with rate 
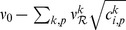
, summing over all independent references indexed by 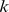. As a result, for two inputs 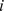 and 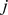 related to a single common reference 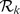, the between-pool cross-covariance is given by
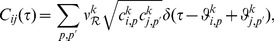
(11)where 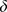 is the Dirac delta function. The correlogram comprises delta peaks at the time difference between all pairs of spikes (indexed by 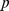 and 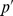, respectively) coming from inputs 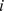 and 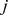. The covariance contributions in (11) from distinct references summate. This method of generating input pattern activity is an alternative to that used in previous studies [8], [9], but it produces similar correlograms.

### Extraction of the principal spectral component

**Figure 5:**
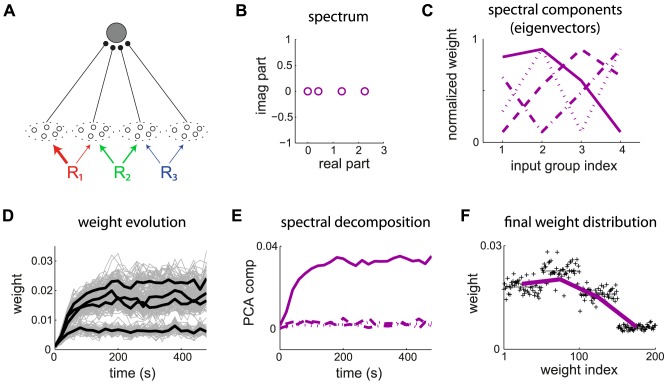
Principal component analysis for mixed correlation sources. (A) The postsynaptic neuron is excited by 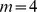 pools of 50 inputs each with the global input correlation matrix 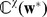 in (13). The thickness of the colored arrows represent the correlation strengths from each reference to each input pool. The input synapses are modified by log-STDP with 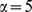. The simulation parameters are given in Table 1. (B) Spectrum and (C) eigenvectors of 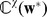. The eigenvalues sorted from the largest to the smallest one correspond to the solid, dashed, dashed-dotted and dotted curves, respectively. (D) Evolution of the weights (gray traces) and the means over each pool (thick black curves) over 500 s. (E) Evolution of the weights 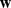 in the basis of spectral components (eigenvectors in C). (F) Weight structure at the end of the learning epoch. Each weight is averaged over the last 100 s. The purple curve represents the dominant spectral component (solid line in C).

This first application shows how STDP can perform PCA, which is the classical spectral analysis for symmetric matrices. To do so, we consider input pools that have multiple sources of correlated activity, which gives within-pool and between-pool correlations. In the example in Fig. 5A, inputs are partitioned into 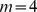 pools of 50 inputs each that have the same firing rate 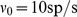. Some pools share common references that trigger coincident firing as described in (11): pools 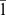 and 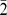 (from left to right) share a correlation reference 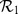 with respective correlation strengths 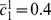 and 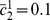 for the concerned inputs; pools 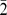 and 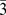 share 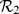 with 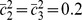; and pools 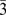 and 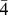 share 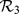 with 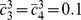. The overline indicates pool variables. All references 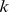 correspond to coincident firing (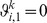) and the rate of correlated events is 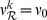. The matrix 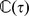 is composed of blocks and given by
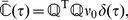
(12)

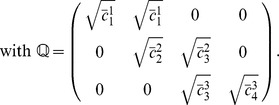



Each row of 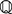 corresponds to a single correlation source here. We further assume that all synapses have identical kernels 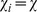. Combining (11) and (3), their covariance matrix 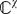 reads
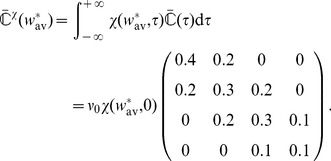
(13)The covariance matrix 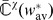 in (13) is symmetric and thus diagonalizable, so it has real eigenvalues and admits a basis of real orthogonal eigenvectors. Here the largest real eigenvalue is isolated in Fig. 5B. The theory thus predicts that the corresponding spectral component (solid line in in Fig. 5C) dominates the dynamics and is potentiated, whereas the remaining ones are depressed. Numerical simulation using log-STDP agrees with this prediction, as illustrated in Fig. 5E. By gradually potentiating the correlated inputs, weight-dependent STDP results in a multimodal weight distribution that can better separate the mean weights of the pools. The final weight structure in Fig. 5F reflects the dominant eigenvector. Despite the variability of individual weight traces in Fig. 5D due to the noise in the weight update and rather fast learning rate used here, the emerging weight structure remains stable in the long run.

### Spike transmission after learning

**Figure 6:**
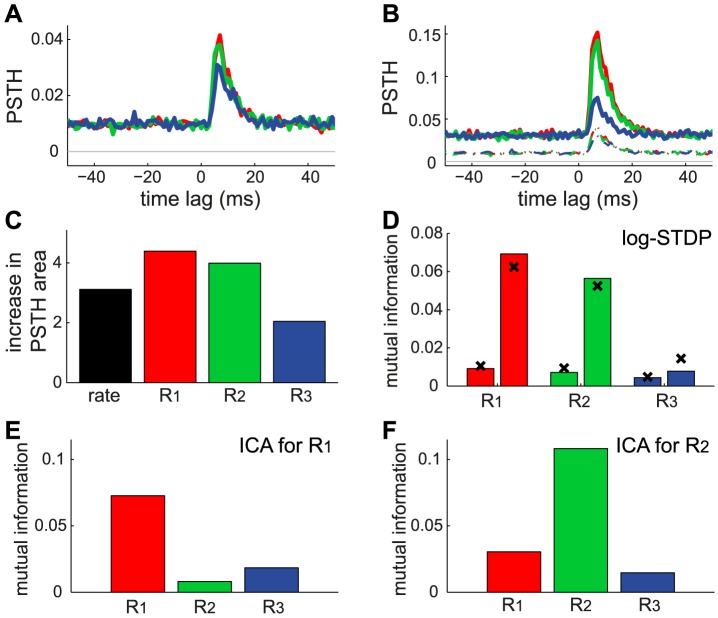
Transmission of the correlated activity after learning by STDP. The results are averaged over 10 neurons and 100 s with the same configuration as in Fig. 5. Comparison of the PSTHs of the response to each correlated event of 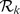 (A) before and (B) after learning for 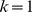 (red), 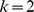 (green) and 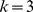 (blue). Note the change of scale for the y-axis; the curves in A are reproduced in B in dashed line. (C) Ratio of the learning-related increase of mean firing rate (black) and PSTHs in B with respect to A (same colors). For each PSTH, only the area above its baseline is taken into account. (D) Mutual information 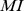 between a correlated event and the firing of two spikes, as defined in (14). For each reference, the left (right) bar indicates 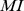 before (after) learning. The crosses correspond to the theoretical prediction using (16) as explained in the text. (E) Example of neuron selective to 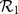 with weight means for each pool set by hand to 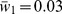; 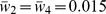 and 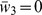. The bars correspond to the simulated 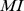 similar to D. (F) Same as E with a neuron selective to 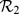 and 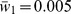; 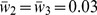 and 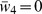.

Following the specialization induced by STDP, the modified weight distribution tunes the transient response to the input spikes. To illustrate this, we examine how STDP modifies the neuronal response to the three correlation sources 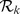 in the previous configuration in Fig. 5. Practically, we evaluate the firing probability during a given time interval of 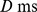 consecutive to a spike from input 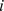, similar to a peristimulus time histogram (PSTH). Before learning, the PSTHs for 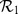 (red), 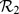 (green) and 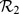 (blue) are comparable in Fig. 6A, which follows because 
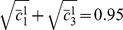
, 
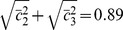
 and 
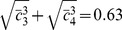
. After learning, pools 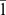 and 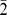 that relate to 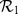 and 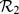 are much more potentiated than pool 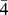 by STDP in Fig. 5F. Consequently, even though pool 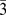 is potentiated and transmits correlated activity from 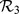, the spike transmission after learning is clearly stronger for 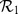 and 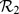 than 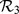 in Fig. 6B. The respective increases of the areas under the PSTHs are summarized in Fig. 6C. The overall increase in firing rate (from about 10 to 30 sp/s) is not supported equally by all 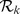.

To further quantify the change in spiking transmission, we evaluate the mutual information 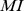 based on the neuronal firing probability, considering correlated events as the basis of information. In this way, the increases in PSTHs are compared to the background firing of the neuron, considered to be noise. In contrast to previous studies that examined optimality with respect to limited synaptic resources [30], [31], we only examine how STDP tunes the transmission of synchronous spike volleys. We define 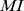 with respect to the event ‘the neuron fires two spikes or more within the period 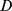
’, denoted by 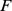; 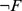 is its complementary. Hereafter, we denote by 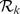 and 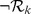 the occurrence of a correlated event and its complementary, respectively The mutual information is defined as
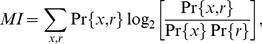
(14)with 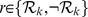 and 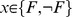. The probabilities are defined for the events occurring during a time interval 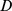. We have 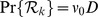 and the realization of 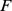 can be evaluated using a Poisson random variable with intensity 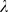


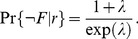
(15)In the above expression, 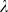 can be evaluated via the baseline firing rate for 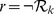 and the PSTHs in Fig. 6B for 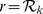. Namely, we adapt (48) and (49) in Methods to obtain
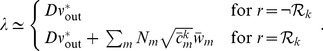
(16)Using the simulation results for the mean 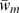 and 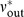 we obtain the predicted values (crosses) for 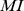 in (14) in Fig. 6D. They are in reasonable agreement with 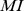 evaluated from the simulated spike trains after dividing the 100 s duration in bins of lengths 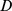. In a clearer manner than with the ratio in Fig. 6C, 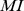 shows that the strong potentiation induced by STDP leads to the reliable transmission (considering that Poisson neurons are noisy) of the correlated events involved in the strong spectral component of 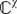, namely 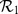 and 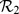, while that for 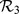 remains poor.

For the input firing rate 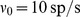 used here, STDP potentiates the weights such that the postsynaptic neuron fires at 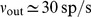 after learning. Because the frequency of correlated events for each source 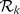 is also 10 times per second, 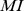 is not so high in our model. Perfect detection for 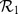 corresponds to firing three spikes for each corresponding correlated event and none ??otherwise. In this case, 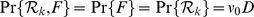, 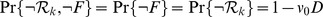 and 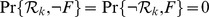, yielding the maximum 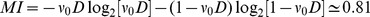. In comparison, for 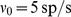 and the baseline log-STDP with 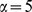 (results not shown), the firing rate after training is roughly eightfold that before learning. Then, 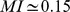 for 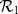 instead of about 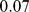 in Fig. 6. For the Poisson neuron especially, high firing rates lead to poor 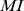 because of the noisy output firing rate. Performance can be much enhanced by using inhibition [9], but we will not pursue optimal detection in the present paper.

### From PCA to ICA: influence of STDP properties on input selectivity

The high neuronal response to both correlation sources in Fig. 6C arises because pools 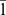, 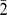 and 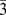 in Fig. 5F exhibit strong weights, in a similar manner to the example with Oja's rule in Fig. 1D. However, it is possible to obtain a much better neuronal selectivity to either 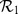 or 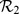, as illustrated in Fig. 6E–F for two distributions set by hand. The corresponding mean weights were chosen such that 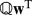 favors the desired correlation source compared to others under the constraint of positive weights; cf. 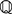 in (12) and 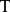 indicates the matrix transposition. We use mutual information as a criterion to evaluate whether kSCA resembles PCA or ICA [33]. Here the analysis of independent spectral component for the postsynaptic neuron means a strong response to *only* one correlation source in terms of 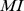.

**Figure 7:**
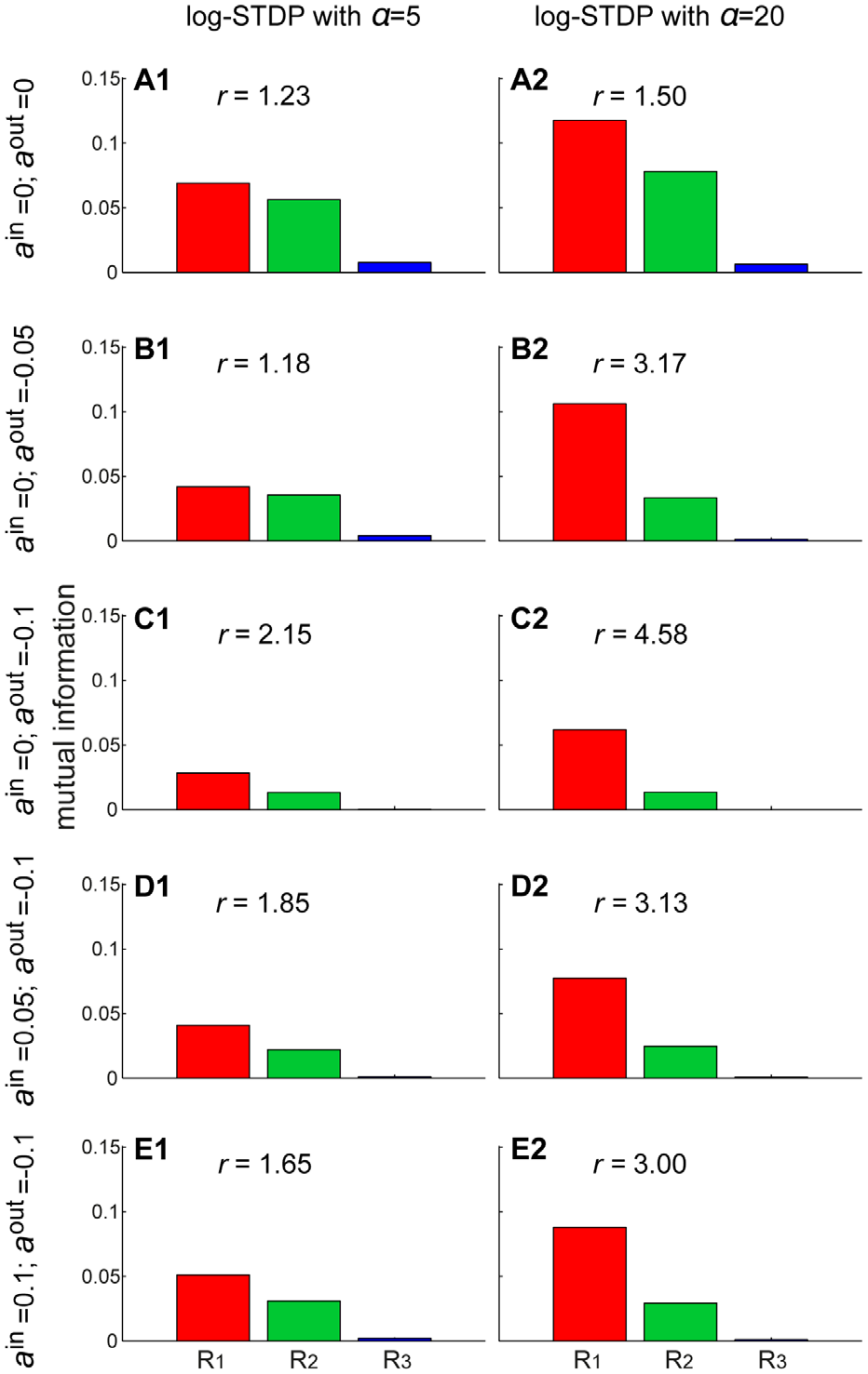
From PCA to ICA. The plots show the mutual information between each correlation source 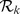 and the neuronal output firing after learning as in Fig. 6D–F. The neuron is stimulated by 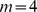 pools that mix three correlation sources as in Fig. 5. The two columns compare log-STDP with different degrees of weight dependence: (1) 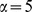 and (2) 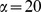 that induces stronger competition via weaker LTD. Each row corresponds to a different combination of single-spike contributions: (A) plain log-STDP meaning 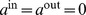 and log-STDP+SCC with (B) 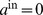 and 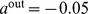; (C) 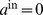 and 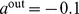; (D) 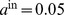 and 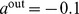; (E) 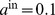 and 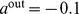. The scale on the y-axis is identical for all plots. The ratio of 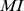 between 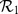 and 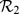 is indicated by 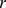.

To separate correlation sources as in Fig. 6E–F, stronger competition between the synaptic inputs is necessary. When tuning the weight dependence of log-STDP toward an additive-like regime, input weights corresponding to the dominant spectral component are more strongly potentiated. This increase results in higher 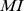 with 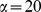 in Fig. 7A2 for both 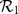 and 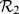, as compared to 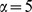 in Fig. 7A1. However, the neuron still responds strongly to 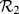 in addition to 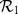, as indicated by the ratio 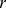 between the respective 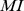. So long as STDP causes the weights to specialize in the direction of the dominant spectral component, pool 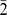 is the most potentiated and the neuron does not isolate 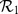. Even for log-STDP with 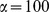 or add-STDP (not shown), we obtain 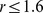. This follows because of the positive input correlations used here. We need a mechanism that causes inputs excited by distinct correlation sources to compete more strongly to drive the neuron. The synaptic competition induced by a negative postsynaptic single-spike contribution 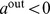 satisfactorily increases the ratio 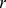 in Fig. 7B–C compared to A (except for B1). One drawback is that the larger negative 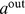 is, the smaller the mean equilibrium weight become, cf. (6). Consequently, even though the ratio 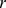 increases, 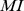 decreases and the transmission of correlations is weakened. To compensate and obtain sufficiently large weights after learning, one can use a positive presynaptic single-spike contribution 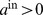. This gives both 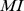 for 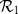 and large ratios 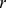 in Fig. 7D2–E2, but not in Fig. 7D1–E1. We conclude that, in order that the neuron performs ICA and robustly selects 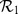, STDP itself should also be sufficiently competitive to obtain robust selectivity, see Fig. 7B–E with 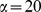 compared to 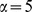. By homogeneously weakening all weights after each output spike in addition to strong STDP-based LTP, only the inputs that most strongly drive the output firing remain significantly potentiated. In other words, 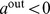 introduces a threshold-like effect on the correlation to determine which inputs experience LTP and LTD. In agreement with our prediction, this “dynamic” threshold becomes more effective for large output firing rates, which only occurs when STDP leads to strong LTP (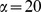). This is reminiscent of BCM-like plasticity for firing rates [49]. Note that we found in simulation (not shown) that using 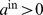 alone did not lead to ICA; this only increases the mean input weights.

**Figure 8:**
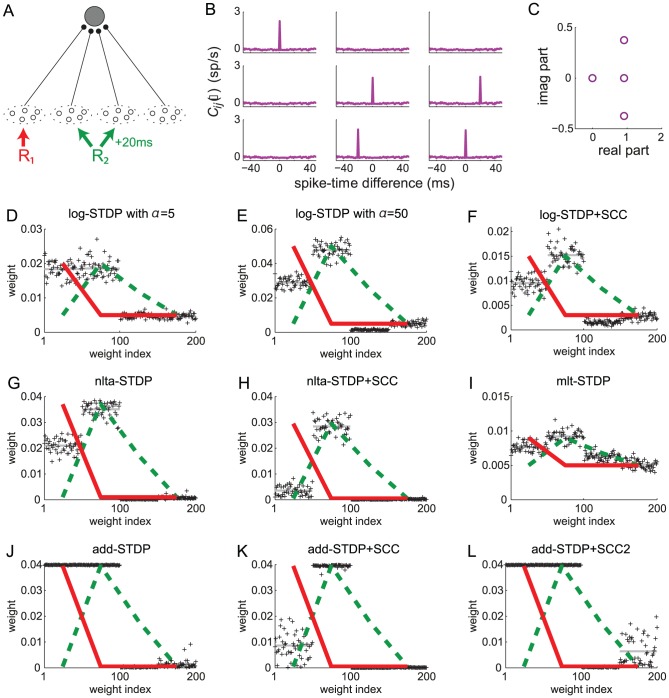
Influence of the STDP parameters. (A) The neuron is stimulated by four pools. From left to right, pool 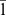 is stimulated by the correlation source 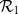 with correlation strength 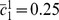 in (11). Pools 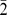 and 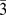 are related to the correlation source 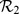 with 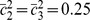; pool 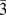 tends to fire 
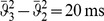
 after pool 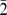. (B) Input cross-correlograms 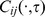 for first three pools described in A. The simulation time is 1000 s and the spike counts have been rescaled by the time bin equal to 1 ms. The peak is predicted by (11). Note the shift of the peak for the cross correlograms between inputs from pools 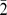 and 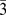. (C) Spectrum of the correlation matrix 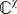 corresponding to B. (D–L) Comparison of the final weight distribution for different STDP models. The two strongest spectral components of the correlation structure in red and green thick lines; they are rescaled between the minimum and maximum weights obtained in the simulation. For STDP+SCC in F, H and K, the single-spike contributions are 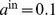 and 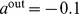. (D) log-STDP with 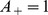, 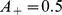 and 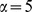; (E) log-STDP as in D with 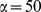; (F) log-STDP+SCC with the same parameters as D; (G) nlta-STDP with 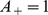, 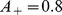 and 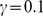; (H) nlta-STDP+SCC with the same parameters as G; (I) mlt-STDP with 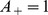, 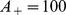 (J) add-STDP with 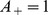, 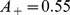; (K) add-STDP+SCC with the same parameters as J; (L) add-STDP+SCC2 with 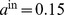 and 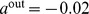.

To further examine the effect of STDP parametrization and assess the generality of our analysis, we examine common trends and discrepancies in the weight specialization for different schemes for weight dependence for plain STDP: log-STDP [16], nlta-STDP [13], mlt-STDP [38] and add-STDP [12]; as well as the influence of single-spike contributions with log-STDP+SCC, nlta-STDP+SCC and add-STDP+SCC [6]. We consider the configuration represented in Fig. 8A where two sources of correlation excite three pools among four. The third pool from the left is stimulated by the same source as the second pool after a time lag of 20 ms. The corresponding spectrum of 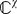 is given in Fig. 8C, leading to two dominant spectral components with equal real part, one for each correlation source. Due to the large imaginary parts of the complex conjugate eigenvalues related to 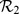, the final distribution in Fig. 8D does not reflect the green component in the sense that pool 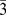 is not potentiated, but depressed. This follows because its correlated stimulation comes late compared to pool 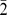. Therefore, the weights from pool 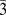 become depressed when the weights from pool 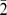 become large. The final weight evolution differ from the initial splitting whereas both weight sets grew (not shown), as expected by the theory. For log-STDP, the weight dependence regulates the number of selected components. Both red and green components are represented in in Fig. 8D, whereas the green component dominates in Fig. 8E. Nlta-STDP can also generate graded distribution as log-STDP does. The synaptic competition in Fig. 8G is comparable to that in Fig. 8E. In comparison, mlt-STDP induces weaker competition, although the asymptotic weights reflect the spectral components in Fig. 8I. On the other hand, add-STDP in Fig. 8J generates a bimodal distribution of weights, which is a thresholded version of Fig. 8D,E or G.

In the case of add-STDP+SCC, the neuronal selectivity is controlled via the equilibrium mean weight 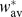 that is constrained by the single-spike contributions 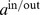 in (6). The situation is more complex for weight-dependent STDP+SCC, as the kernels 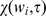 is modified by as the weights evolve. Nevertheless, similar effects were observed in simulations. For log-STDP+SCC (Fig. 8F) and nlta-STDP+SCC (Fig. 8H), the qualitative profile of the final weights is similar to that for plain STDP, with the additional competition induced by 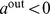 that depresses pool 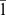 and favors 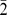, as was described in Fig. 7. In the case of add-STDP+SCC, the instability of the dynamics leads to more sensitivity to the single-spike contributions. With 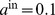 and 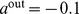 in Fig. 8K, only the weights from pool 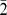 are potentiated at the end of the learning epoch. However, with 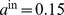 and 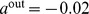 in Fig. 8L, the competition is weakened and all weights from pools 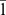 and 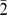 are potentiated, in agreement with the theoretical prediction. Interestingly, some weights from the uncorrelated pool 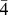 are mildly potentiated, whereas those from the positively correlated pool 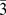 are more strongly depressed toward zero because of the time lag associated to 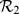.

### Influence of the postsynaptic response

**Figure 9:**
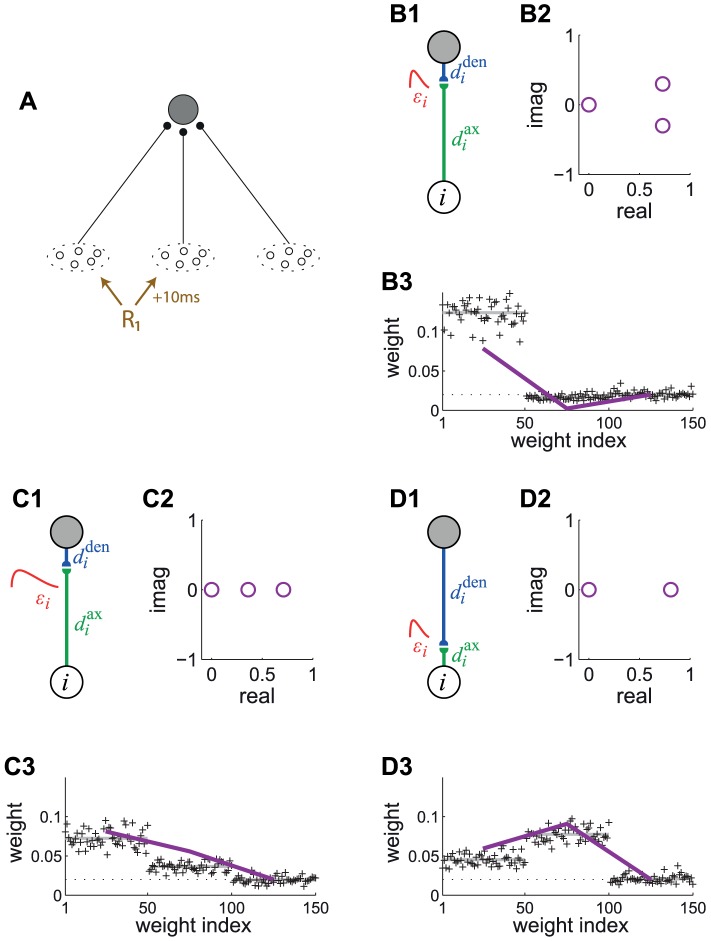
Influence of the PSP response on the kernel 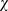 and the resulting weight structure for log-STDP. (A) The neuron is stimulated by 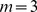 input pools. The first two pools have the same reference for correlations with strength 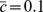 and pool 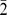 tends to fire 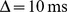 after pool 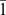. Pool 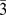 has no correlation. For all inputs, the firing rate is 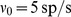. (B) Short PSP response with 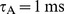 and 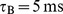, as well as purely axonal delays 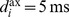. (C) Long PSP response with 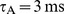 and 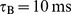, as well as purely axonal delays 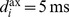. (D) Short PSP response with 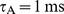 and 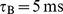, as well as purely dendritic delays 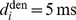. For each configuration, we present (1) a schematic diagram of the synaptic parameters, (2) the eigenvalues of 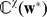 and (3) the resulting weight specialization. As in Fig. 5, the purple curves represent the expression in (10). The dotted horizontal line indicates 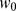, the equilibrium weight for log-STDP. Two eigenvalues are roughly equal to zero in D2.

Now we examine how the postsynaptic response affects the weight competition. This turns out to be particularly important when the correlograms have a temporal extension, that is, richer than just narrowly correlated inputs with a peak at 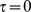. We consider the configuration in Fig. 9A where inputs from the pool 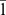 tend to fire a time lag 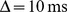 before those of pool 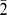. Namely, correlation is generated following (11) using a reference 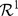 with 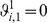, 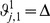, and 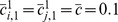. Pool 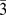 has no correlation. The matrix 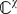 in (3) averaged over pools is not symmetric:
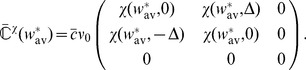
(17)


Following (4), the PSPs and delays affect the kernel 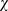 (here identical for all synapses), hence 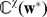 and the resulting weight selection. In Fig. 3C, the same STDP learning window is combined with different PSP kernels and synaptic delays. We first use the baseline parameters in Fig. 9B1: a rise constant 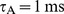 and a decay constant 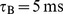 for the PSP kernel and purely axonal delays 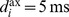. They correspond to the blue curve in Fig. 3C. In this case, the matrix 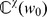 may be rather antisymmetric (outside its diagonal):
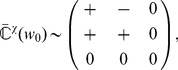
(18)cf. the values of the blue curve indicated by the arrows in Fig. 3C. The eigenvalues are represented in Fig. 9B2. This indicates that the (correlated) pool 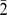 fires “late” with respect to pool 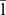, from the point of view of STDP. It follows that the second pool is depressed while the first pool is potentiated, as illustrated in Fig. 9B3.

In contrast, a different weight selection occurs for the same axonal delays, but longer PSP time constants in Fig. 9C: 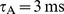, 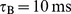 (the purple curve in Fig. 3C); as well as dendritic delays 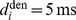 with the same short PSP time constants in Fig. 9D (green curve in Fig. 3C). In both cases, this follows because 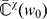 has the following form:
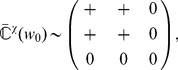
(19)which is “more” symmetric compared to Fig. 9B, and thus does not depress the late pool. The change in 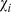 affects the spectrum, which results in the potentiation of both correlated pools 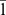 and 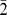, as illustrated in Fig. 9C2 and D2. For the case of a delay of the dendritic delay in Fig. 9D3, the late pool 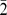 is more strongly potentiated than the early pool 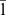 as 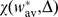 corresponds to the peak of the kernel, cf. the right arrow and the green curve in Fig. 3C. This illustrates that the effect of pool 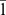 on the output firing felt at the synapse (i.e., after twice the dendritic delay 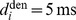) coincides with the firing of pool 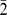, namely 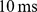 after pool 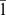.

## Discussion

We have demonstrated how the STDP dynamics reflect the spectral properties of temporal correlations conveyed by input spike trains. The present analysis links the physiological properties of STDP, such as its learning window and weight dependence, to function in terms of spiking information. It sheds light on recent numerical studies [7], [29] that used STDP to separate correlation sources, thus performing ICA. Such spectral decomposition capabilities appear to be the inherent algorithm of STDP. We find that, for a neuron with linear input-output response, excitatory pairwise STDP *alone* performs PCA-like computations. Weight-dependent STDP that induces both graded and robust LTP generates a rich representation of the input correlation structure. However, an additive-like weight dependence is not sufficient for ICA in general. In order to achieve ICA, STDP requires an additional homeostatic mechanism. Here we have used LTD triggered by single output spikes that prevents all weights from growing and results in enhanced competition between correlation sources.

### Input configuration and spectral components

For pairwise STDP, the weight dynamics can be predicted provided the firing rates and pairwise cross-correlations are well defined. The corresponding expressions (35) and (36) in Methods highlight the separation of timescales between rate-based and spike-based effects, which is determined by the learning window function 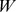. Spike-time correlations arise when coordinated firing between neurons is consistently repeated over time, such as a repeating spatiotemporal pattern embedded in random spiking activity and peaked PSTHs in experimental data. In the correlation structure induced by such pattern presentations, strong spectral components correspond to dense and peaked clusters of pattern spikes, in a similar fashion for both spike coordination and rate covariation [9]. Our framework can account for a rich variety of input configurations, in particular, stimuli that were used with STDP for categorization and/or representation in previous studies [8]–[10], [50]–[52], as well as recently proposed elaborate input configurations [46]–[48]. Time-varying signals can also generate significant spike-time correlations and thus weight specialization (Text S2 and Fig S2).

### Kernel spectral component analysis (kSCA) of input spike-time correlations

The present framework aims to provide a unified description of the STDP dynamics for the many configurations that have been used in previous studies. Following the observations by Gerstner and Kistler [25, Ch∼11], STDP potentiates and depresses weights depending on the spectral components of 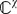. This matrix embodies the STDP-specific effects and is determined by the input correlation structure 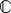 and kernels 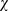. The kernels are determined by the STDP learning window and PSP responses, cf. (4). In a sense, the cross-correlograms in 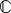 are “seen” by the neuron through the kernels 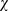. This is especially important when input correlograms have a temporal extension (Fig. 9) or when the shape of the STDP learning window function 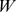 varies across synapses. When using long timescales for PSPs with usual time constants for the learning window 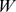, the matrix 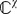 tends to be symmetric and the PCA performed by STDP can result in slow-feature extraction [27]. Another point is that the input correlation structure as a *whole* determines for the weight specialization. In Fig. 8L for example, uncorrelated inputs are not as depressed by STDP as some positively correlated inputs. The present study has focused on Hebbian STDP for excitatory synapses (Fig. 2C), but the same framework can be used for any arbitrary learning window 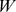, as well as the case of plastic inhibitory synapses [53]. A neuron can thus generate elaborate representations of the stimulating inputs in its weight structure, which illustrates the versatility of STDP.

#### Relationship to Oja's rule

When the input configuration corresponds to mixed instantaneous correlations, STDP alone can perform PCA on the correlation strengths (Fig. 5). In this way, STDP can be seen as an extension of Oja's rule [24], as was suggested by van Rossum et al. [26]. There are several important differences, though:

- Oja's rule relies on rate-based information, which implies a symmetric cross-correlation matrix between inputs and thus performs PCA (Fig. 1). STDP, however, is based on the spike-time correlograms contained in 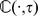. The matrix 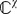 is determined by the kernels 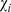 (Fig. 3) and may thus not be symmetric, especially for the usual temporally Hebbian learning window 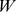. This implies richer weight specialization via the interaction with the neuronal parameters (Fig. 9).
- When the eigenvalues have large imaginary parts (Fig. 8), the final weight distribution may not reflect the initial weight splitting. Nevertheless, the weights that win the synaptic competition, in the sense of being eventually most strongly potentiated, are satisfactorily predicted by (10).
- In addition to the first Hebbian term in (1), the second term in Oja's rule leads to a specific constraint that drives the weights toward the principal spectral component. When STDP performs PCA-like computations, adequate weight dependence resulting in graded LTP results in a better representation of the principal component (Figs. 5 and 8).
- For STDP, the resulting neuronal selectivity is determined by the weight dependence, as well as the additional homeostatic mechanisms. This allows flexibility in tuning the learning process. For example, STDP can also switch from PCA to ICA, as will be discussed in more depth below. In contrast, more than one spectral component of 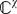 can be selected when the competition is not too strong (Fig. 8D). On the other hand, Oja's rule requires several neurons to extract several spectral components, as each neuron only selects a single component.

#### Influence of weight dependence

When STDP performs PCA, a desirable outcome is a fine representation of the main spectral component of the input covariance in the weight structure. When the weight evolution is consistent with the initial splitting, the final weight distribution reflects the principal component in (10), as illustrated in Figs. 5F and 9. The key is a graded potentiation of correlated inputs as induced by log-STDP [13] or nlta-STDP [16]. This functional property of the experimentally observed weight dependence complements previous conclusions about its role in regulating the synaptic competition and shaping the weight distribution [13], [15], [16], [38], [39]. To obtain effective weight specialization, STDP should be parametrized in an additive-like regime. However, purely additive STDP often leads to a bimodal distribution of synaptic weights, which may not reflect the complexity of the input structure. In addition, we have observed that add-STDP can lead to unstable synaptic dynamics over an extended learning epoch. Figure S1C provides an example of quasi-periodic evolution of the synaptic weights when 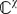 has dominant eigenvalues with large imaginary parts. Even a slight dose of weight dependence appears sufficient to introduce stability in the weight dynamics in this case (Fig S1D), which agrees with the existence of a fixed point predicted by our analysis.

#### From PCA to ICA

An important conclusion of our results is that kSCA performed by *plain* STDP relates to PCA, but differs from ICA. For example, the dominant component of the input correlations in Fig. 5 mixes the correlations from 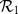 and 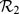. So long as STDP potentiates the weights in that “direction”, the trained neuron does not become selective to only *one* correlation source. However, additional constraints on the weight dynamics can disrupt this scheme. As shown in Fig. 7, when the additional competition induced by 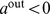 [6] is sufficiently strong, the neuron can become selective to a single correlation source by tuning its positive synaptic weights. This results in a winner-take-all situation for the strongest correlation source involved in the dominant spectral component. This competitive effect complements the homeostatic regulation on the mean weight by the pre- and postsynaptic single-spike contributions [35]. The neuronal selectivity in Figs. 6 and 7 is measured using the mutual information between input correlated events and output firing. This provides a suitable criterion to discriminate between PCA and ICA [33] and has been used to evaluate the performance of STDP in extracting information within noisy spike trains [30]–[32].

There exist configurations where PCA and ICA coincide, for example, when each spectral component (eigenvector) is associated with a single correlation source (Fig. 8A). Then, the selection of *one* eigenvector actually results in ICA. In such cases, pairwise STDP in a competitive (additive-like) regime can lead to a symmetry breaking, namely segregation between similar eigenvalue of 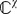 [13], [16], [17]; see also Fig. 8E compared to D. Therefore, we have used mixed correlation sources to investigate more carefully whether kSCA resembles PCA or ICA (Fig. 5), in a similar manner to Fig. 1 where the correlation sources overlap.

One issue with ICA in our model is that the performance crucially depends on the values 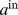 and 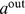. For distinct input configurations, these values may have to be adjusted. Two opposing effects are operating here. First, The competition due to 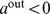 that brings ICA becomes stronger for increasing input correlation strength if the neuronal firing rate becomes stronger. Second, large negative values for 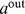 prevent the weights from being strongly potentiated, which leads to a low output firing rate. Further work is necessary to understand this interplay in more depth. An alternative to 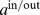 to regulate the mean weight is a homeostatic weight scaling [38], [54]. The precise nature of such a scaling critically affects the neuronal selectivity. When combined with rate-based Hebbian learning, subtractive normalization enables symmetry breaking, whereas multiplicative normalization leads to a form of PCA [55]. Previous studies that managed to perform ICA using STDP used a homeostatic weight scaling that normalizes the mean weight [29]. In an abstract learning model, Xu et al. [56] have demonstrated how such weight normalization constraints can cause the same update rule to switch between PCA and other algorithms such as ‘k-means’, i.e., grouping input pools at distinct levels of potentiation (cf. Fig. 8J–K with the first two pools).

To achieve ICA with arbitrary input configurations with automatic tuning, adaptative nonlinearities in the neuronal response have been successfully used [29]. Such a nonlinear neuronal response captures higher-than-second-order correlations in a similar fashion to previous studies using rate-based learning [57]. Intuitively, superlinear PSP responses boost the competition between the weights, which prevents the output neuron from strongly responding to independent correlation sources. Likewise, STDP models relying on triplets of spikes can use such higher-order statistics to separate correlation sources [58]. Last, we have only considered positive weights here. The weights may be eventually potentiated or depressed compared to the mean equilibrium value for uncorrelated inputs (this difference is the equivalent of positive/negative weights in PCA in machine learning). Only significantly stronger weights transmit correlation patterns efficiently, whereas weaker weights hardly drive the postsynaptic neuron. Although ICA-like specialization can be achieved under the constraint of positive weights (Fig. 6E–F), inhibition can enhance the input selectivity when it strongly suppresses the transmission of certain correlation patterns [59].

### Extension to more elaborate STDP and neuron models

The present study has focused on STDP contributions up to the second order (pairs of pre- and postsynaptic spikes) and the learning dynamics that arise from the effect of pairwise spike-time correlations. This means that higher-order correlations only play a role via their collective second-order effects. In contrast, triplets or bursts of spikes can significantly modulate the weight updates in other models [40], [60]. The model proposed by Appleby and Elliott requires multispike interactions (i.e., higher-order correlations) for synaptic competition to emerge [41]. More elaborate STDP models also present advantages for spike computation and/or reproducing experimental data [7], [31], [34], [40], [61], [62]. In addition to the effect of spike-time correlations considered here, some of these models are sensitive to firing rates. Likewise, when spike pairs contributing to STDP are restricted (whereas all pairs are included in our model), the equilibrium mean weight depends on the input firing rates [4], [5] and the balance between spike and rate effects is affected. Our results are expected to hold at least partially when pairwise effects dominate the STDP dynamics. Extending our study is left for subsequent work, but making use of higher-order correlations appears promising to perform ICA [58]. Although our STDP update incorporates noise, our analysis neglects it and assumes that the weight drift (i.e., mean change or first stochastic moment) dominates the dynamics. In extreme cases, a fast learning rate can compromise the stability of the emerged weight structure [14].

The present analytical study is based on the “linear” Poisson neuron, which allows a tractable analysis. Its stochastic firing mechanism generates rather noisy and unreliable spike trains compared to deterministic neuron model where the stochasticity arises from the inputs, e.g., integrate-and-fire neurons. Similar weight dynamics for both models have been demonstrated previously for slow STDP learning [6], [13]. As mentioned above, a nonlinear firing response may be useful to perform ICA. In order to go beyond the linear input-output regime for integrate-and-fire neurons [63], it is necessary to study how the neuron model shapes the input-output covariance; see (32) in Methods. In most neuron models, larger excitatory weights induce stronger input-output correlations for correlated inputs. This results in a positive-feedback loop for learning, which is captured by the Poisson neuron model. Dendritic integration of synaptic inputs are expected to bring interesting nonlinearities to the kernel 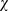 defined in (4). Moreover, depending on regional competition between and within dendritic branches [64], [65], different components can be represented in distinct areas of a single neuron. Including such refinements opens promising ways to understand spike-based computations.

### Implications for spiking information processing in neuronal networks

Finally, our results support the idea that neurons equipped with STDP can operate as self-adapting filters that process information based on the transient firing response of neurons. The input-output spike-time covariance (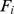 in our model) is simply the average of the transient response over all input statistics. STDP tunes these input-output correlations based on the input cross-correlation structure (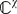). Extending previous results focusing on a single correlated pathway [12], Fig. 6 illustrates the modification of the transmission of coincidentally spiking activity using mutual information as a measure of signal-to-noise. This view is consistent with the hypothesis that the coordinated activity of cell assemblies can serve as a basis for the neuronal code [66]. In a more general scheme, spiking information should also consider the detailed shapes of the correlograms, not just their integral value as here. Because of the temporal dimension, coding using correlations appears richer than rate-based coding, as was observed in experiments [67]. Propagation of coordinated transient spiking activity, which can be seen as a generalization of PSTHs or spike patterns [9], appears suitable for coding/decoding and naturally interacts with STDP. Depending on the more or less peaked shapes of the corresponding correlograms, the neurons may operate in either closer to a spike-based or a rate-based regime; these two forms of neuronal coding in feedforward networks are actually the two sides of the same coin [68]. Here correlations involve multiple input spike trains and all neurons belonging to the same assembly exhibit pairwise correlograms that have “coordinated” shapes, in a similar manner to cliques in graphs. Although a formal quantification has yet to be defined, the information in 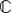 can intuitively be understood in terms of the diversity and arrangement of cross-correlograms. The kernels 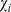 then define a “similarity measure” on matrices 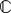: the respective shapes of the correlograms and kernels determine the effective strength of spectral components.

In a network, heterogeneity in the synaptic properties (PSP response and delays) and STDP learning windows leads to distinct kernels 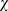 among the synapses, so neurons can extract different components from a common input correlation structure. This can be used by (inhibitory) STDP to extract the frequency of rhythmic neuronal activity [53], which has been observed in many brain areas. Large inhomogeneities are expected to affect the weight specialization for oscillatory signals [10], [11], [45], [69]. They may also play a role in encoding of slow signals at the shorter timescale of STDP [70], [71]. Likewise, partial input connectivity allows neurons to see only part of the same global input structure, leading to differentiated specialization that may represent many spectral components. However, further developments are necessary to extend this analysis to the case of recurrent connections, which constrain the correlation structure [19], and incorporate possibly plastic inhibitory connections. This theory aims to better understand how neurons can process spiking information in a distributed fashion [72]. Interesting applications have been proposed recently [73], [74]: STDP can preprocess temporal signals within a recurrently connected network that act as a (huge) reservoir of functions of the inputs, which enhances the performance of the so-called liquid state machine [75]. Cessac et al. also showed that STDP can change the network activity such that observables (e.g., firing rates, spiking synchrony) obey Gibbs distributions [76]. Together, these efforts will hopefully lead to novel interpretations on how neurons can process spike trains.

## Methods

After the presentation of the STDP models, the following sections detail the derivation of the learning equation (2), which is analyzed in Results. The spike-time covariances of presynaptic spike trains, which is the crucial input information for the kSCA algorithm, are formally defined in a later section. Conditions on the existence of a stable fixed point for the weight dynamics are then derived. Finally, the neuronal response to input correlations is calculated in a simple case, which is used to evaluate theoretically the change in mutual information in Results.

### Phenomenological model of pairwise weight-dependent STDP

Pairs of pre- and postsynaptic spikes, as well as single spikes, completely determine the contributions to STDP. This choice has limitations compared to more elaborate models that include, for example, triplets or bursts of spikes in their analysis [40], [69] or models for which pairwise correlations do not generate competition [41]. This choice allows us to focus on the next stochastic order after firing rates (first order) while keeping the analysis tractable.

For a pair of pre- and post-spikes whose effects reach the 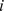
th synaptic site at times 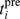 and 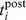, respectively, the weight 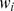 is modified by the following additive terms
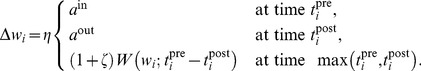
(20)In general, we assume that the STDP-specific update 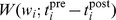 depends on the current value of the weight 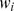 [13], [38], [39], [42], in agreement with experimental evidence [37]. This weight dependence alone can stabilize the weight distribution for ‘plain STDP’, i.e., without single spike contributions. However, in the absence of weight dependence, single-spike contributions 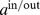 are necessary to enforce partial stability on the weights, namely homeostasis on their mean [6], [35], [36]. Note that both mechanisms can also be successfully used together [15]. We will refer to the case where 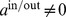 as ‘STDP+SSC’, in contrast to ‘plain STDP’ (or ‘STDP’ alone when there is no possible confusion) for 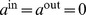. The second case is often regarded as more biologically plausible for excitatory STDP and will be the focus on this work. Although their effect is not considered in detail, the weight update in (20) involves a learning rate 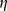, which determines the speed of learning, and variability in the pair-specific contribution, which is modeled by the white-noise random variable 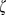 that has zero mean and variance 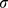.

As mentioned above, the contribution specific to spike pairs depends on the relative timing of pre- and postsynaptic spiking activity felt at the synaptic site. For the synapse 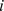 described in Fig. 2B, a pulse fired by the presynaptic neuron 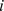 at time 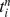 and a pulse fired by the postsynaptic neuron at time 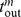 correspond to
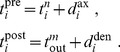
(21)Typically for excitatory STDP, 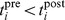 leads to potentiation (LTP) and, conversely, 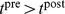 to depression (LTD). Thus, 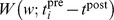 can be expressed as
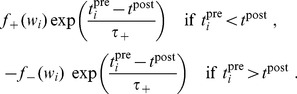
(22)Here decaying exponentials are used for illustration purpose.

We compare several schemes for the weight dependence that is defined by the scaling function 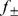:

- Our recently proposed ‘log-STDP’ model [16] has a sublinear LTD (log-like saturating profile for 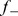) can produce to long-tail (lognormal-like) distributions of synaptic weights. Here we use
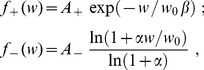
(23)where 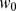 is a reference weight, 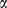 controls the saturation degree of LTD and 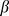 the (slow) decay of LTP when the weight 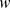 increases.
- The ‘nlta-STDP’ model proposed by Gütig et al. [13] corresponds to:
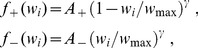
(24)where 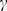 scales from additive STDP with 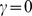 [6], [12] to multiplicative STDP with 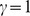 that has a linear dependence for both LTP and LTD [39]. The “soft” bound 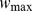 is enforced on the weights. In numerical simulation, we will use 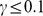 to obtain sufficiently strong competition between the synaptic weights.
- The special case of (24) with 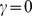 is weight independent, namely, additive STDP with 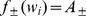, will be referred to as add-STDP.
- The ‘mlt-STDP’ model proposed by van Rossum et al. [13] corresponds to:
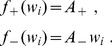
(25)

Baseline parameters used in numerical simulations are recapitulated in Table 1.

### Capturing the weight dynamics

The analysis is constrained to a single neuron excited by external inputs indexed by 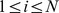. The spike trains of the neuron and external input 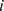 are denoted by 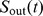 and 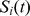, respectively. We use a previously developed framework [6], [36] to analyze the effect of weight-dependent STDP on the input plastic weights 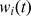.

The tractability of the present analysis relies on the condition that both the firing rates 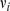 and covariances 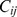 are quasi-invariant with respect to time 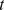 (but not for the time lag 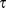). We assume that learning occurs sufficiently slowly compared to the other neuronal mechanisms (i.e., PSP time constants and delays) and that the noise 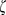 is not too strong, such that the drift (or first stochastic moment) of the weight dynamics essentially determines the emerging structure [14], [77]. Under this “adiabatic” assumption, the weight evolution can be described by
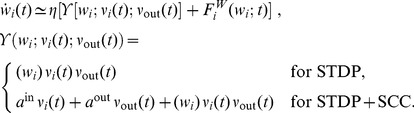
(26)The weight update in (26) is the summation of two additive contributions. First, the rate-based contributions embodied by 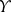 involve the time-averaged firing rates 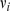 and 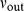 for input 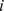 and the neuron, respectively, cf. (35). For weight-dependent STDP, it involves the integral value of the learning window (as a function of the current weight)
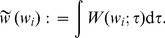
(27)Second, the covariance coefficient 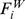 incorporates the effect of the STDP on the time-averaged spike-time covariance 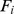 between the neuron and input 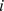:
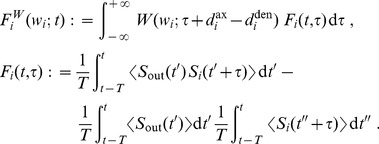
(28)Note that the noise 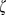 does not play a role in the weight drift evaluated here. In the Results section, we will show that the predicted weight specialization is valid even for a medium level of noise in STDP. In order to analyze the learning equation (26), we need to evaluate the neuronal firing rate 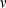 and covariance coefficients 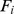 in terms of the input parameters. For this purpose we need to specify the neuronal firing mechanism.

### Poisson neuron model

In the Poisson neuron model [6], [13], [14], [19], [36], the neuronal spiking mechanism is approximated by an inhomogeneous Poisson process driven by an intensity function 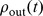 in order to generate an output spike-time series 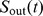. A presynaptic spike from input 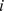 induces a variation of 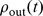 referred to as the postsynaptic potential (PSP), which is determined by the synaptic weight 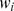, the kernel function 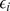, and the sum of the axonal and dendritic delays 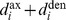. We require 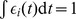 and, in order to preserve causality, 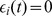 for 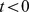. For illustration purposes, we choose a double exponential function for all 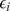:
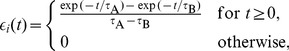
(29)with rise and decay time constants 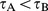, respectively. The “soma potential” 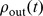 sums the PSPs for all input spike times 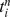


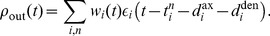
(30)


Following (30), we obtain the consistency matrix equations for the firing rates and spike-time correlations:
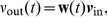
(31a)

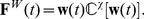
(31b)Here 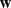 and 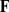 are 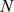-row vectors and 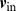 a 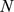-column vector, whose elements are 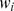, 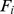 and 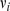, respectively; bold capitals will be used for row vectors and bold lower-case characters for column vectors. The 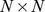 matrices 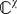 have elements that correspond to pairs of inputs 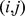:
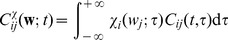
(32)is reproduced in (3). Note the respective roles of indices 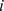 and 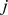. The input covariance 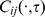 is assumed to be quasi-independent of time 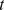, so 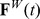 in (32) only depends on 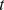 through the weights 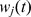, which slowly evolve due to STDP. The kernel functions 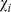 in (33) describe the interplay between STDP and the postsynaptic response kernels 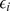 that affects the weight dynamics:
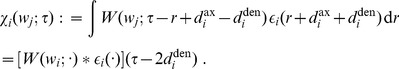
(33)The convolution indicated by 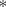 concerns the variable 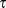. This equation is reproduced in Results, cf. (4). This means that the postsynaptic response crucially determines the effect of synaptic plasticity [19], [27]. In particular, the dendritic delay 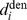 plays a distinct role compared to the axonal delay 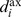 in that it shifts the kernel 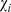 as a function of 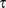 to the right, namely implying more potentiation for 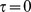. Because of the weight dependence, the kernel is modified via the scaling of both potentiation and depression for 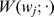 when the strength 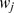 evolves, as illustrated in Fig. 2C. The combination of (26) and (31) leads to (2), where the dependence over time 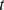 is omitted.

### Description of input spiking structure

The following expressions allow us to deal with general inputs while at the same time satisfying the requirement for mathematical tractability. We denote by 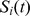 the spike train (Dirac comb) of input 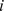. The corresponding time-averaged firing rate is defined as
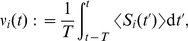
(34)and, for a pair of inputs 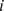 and 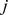, the spike-time cross-covariance 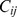 is given by
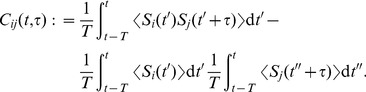
(35)


A double averaging is used in the above definitions:

- an ensemble average over the randomness (because the input spike trains will be generated using stochastic processes) denoted by the angular brackets 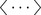 and
- a smoothing over a period of duration 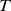, chosen to be larger than the timescale of neuronal mechanisms, but smaller than the learning rate of STDP.

The separation of timescales implies that only correlations convey fast spiking information, whereas firing rates imply low-pass filtering. The covariance 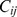 in (36) slightly differs from our previous framework [15], [36]. It is actually the sum of two contributions: the stochastic covariance between the spike trains averaged over 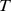, which relates to ‘spike coordination’:
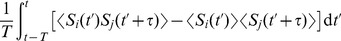
(36)and the temporal covariance of the underlying rate functions, which we refer to as ‘rate covariation’:
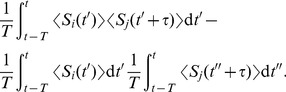
(37)For inputs generated using doubly stochastic processes [25], a double ensemble average has been used in a similar fashion to the combination of ensemble average and temporal integration here. With our convention, the graphical interpretation of correlogram 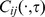 is that peaks for positive values of 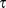 (right side) indicate that input 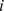 tends to fire earlier than 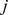. For oscillatory inputs, if the closest peak to 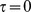 is on the right side, 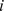 is phase-advanced compared to 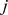.

### Conditions ensuring the existence of a stable fixed point for weight-dependent STDP

Here we examine the conditions under which there exists at least one fixed point such that (2) for plain STDP vanishes for all coordinates 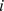, namely
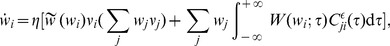
(38)where 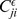 denotes the convolution of the correlation with the PSP kernels 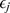, reorganizing (33). We make a couple of assumptions here:

- The weight dependence is such that the LTD side of 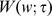 vanishes when 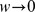, whereas the LTP side vanishes for 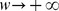. In particular, this implies that 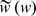 in (27) is a decreasing function of 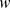 and has a zero 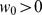 where LTD balances LTP. This property is satisfied by both log-STDP and nlta-STDP.
- The inputs have positive correlations, meaning that 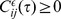 for all pairs 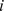 and 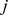.

We define for sake of simpler notation the following functions such that (39) reads 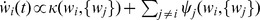 with
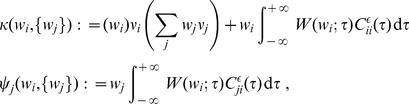
(39)where 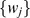 denotes the whole set of weights.

For each given 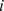, the sign of the first term in 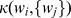 is given by 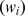 alone and does not depend on 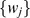:
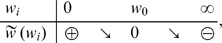
(40)where the circled signs indicate positive and negative values. The second term is zero for 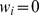 and for a sufficiently large 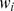, it becomes negative (or barely positive) with the assumptions that LTP vanishes and 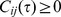. For all 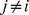, the sign of 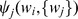 is given by 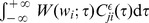 and 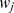 scales its modulus linearly:
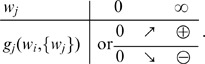
(41)Taken together, we have for an arbitrary small 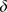


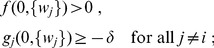
(42)and there exists a set of constants 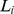 such that
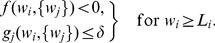
(43)These are sufficient conditions to prove that the expressions in (39) taken for all 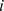 have at least a global fixed point.

We first examine the illustrative case of 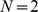 weights. For any fixed 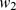, the expression of 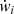 in (39) satisfies the two properties of being positive on the axis 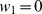 and becomes negative for large 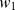, following (43) and (44). Consequently, for all 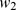, there is at least one zero of 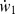 as a function of 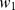, and this zero is strictly positive and smaller than the upper bounds 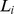. Moreover, the expression in (39) for 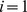 is continuous with respect to both 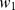 and 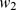, so the zeros form a continuous curve. Reciprocally, by inverting the indices, there is a similar zero for 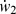. Because of continuity, there is at least one intersection point for the sets of the zeros as in Fig. 4A, which nullifies 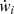 for 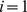 and 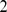.

In the general case of 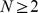 weights, the same properties in (43) and (44) ensure that, for each given 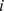, (39) is positive on the hyperplane 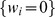 and negative on 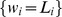. It follows that there is at least one zero for each 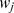, 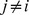. Thus, the continuous surface that contains the zeros of (39) for a given 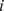 contains a manifold of dimension 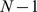. In the 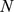-dimensional hypercube 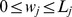, all such manifolds for 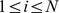 have at least one intersection point, since the “constraint” for being on the 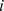-th manifold only concerns 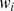. On the non-empty intersection set, all derivatives 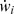 vanish, meaning it consists of the fixed point(s) for the weight dynamics.

The structure of these manifolds is actually simple and allows us to determined to the global stability of the fixed point(s). For each 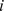, the corresponding manifold separates the hypercube 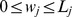 into two subspaces. On the side containing 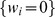, we have 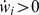, whereas on the other side 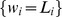, 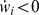. Each manifold is thus a global attractor for the coordinate 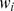, which guarantees global stability of their intersection set. The arrows in Fig. 4 illustrate the derivatives of 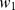 and 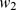, which drive 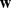 to its fixed point there.

Now, for negative correlations, (43) or (44) may not hold anymore and the zero of (39) may become negative or even not exist for some values of 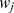. There is then no guarantee of a realizable global fixed-point, as illustrated in Fig. 4B. The analysis in this case will not be pursued here.

A similar demonstration applies for STDP+SCC when 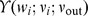 is positive for 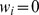 and decreases with 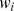. This is the case when 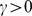, for which LTP and LTD vanish at the upper and lower bounds enforced on the weights, respectively, in addition to 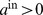. With the further condition 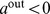 that ensures a fixed-point for the mean weight, the equivalent to 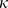 decreases when the output firing rate 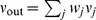 increases. Putting it all together, the existence of a fixed point is ensured for output firing rate that are not too high (and positive correlations).

### Relationship between the final weight distribution and the initial weight splitting

In the early period of the weight evolution, we can approximate the weight vector as proportional to the dominant eigenvector(s). Firstly, we consider the case of a single dominant eigenvalue, namely 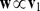. The spike-based term of (2) can be rewritten
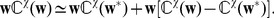
(44)Decomposing 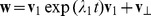 for some initial factor 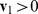 and 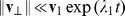, the first term of the rhs 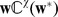 is dominated by its component 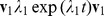. This follows because 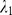 is the largest eigenvalue. Now we further assume that the weight dependence is “weak” with respect to 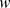. By this, we require the second term of the rhs above to be dominated by the first term. Together, this means that the vector elements of 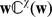 are ordered as those of 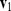. The fixed point of the dynamics in (2) can be approximated by
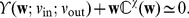
(45)We assumed earlier that the weight dependence is such that the 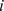-th component of 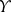 is a decreasing of 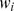. The implicit relationship in (46) indicates that the fixed point of 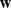 is given by the reciprocal function to 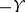 applied on 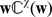, which has its vector elements sorted in the same order as 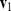 as we just explained. In other words, 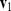 is expected to reflect the final weight distribution under the mentioned assumptions for a single dominant eigenvalue.

In the case of two complex conjugate dominant eigenvectors, a large imaginary part for 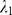 implies a strong rotation-like evolution even at the early stage: 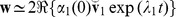. In this case, the equilibrium weight distribution may significantly differ from the initial splitting in the direction of 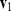. As a illustrative example, we consider two weights 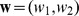 with
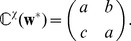
(46)


This expression corresponds to the cases of time-lagged correlated inputs in Figs. 8 and 9. When 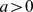, 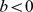 and 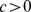, 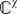 has complex conjugate eigenvalues 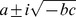. Larger absolute values for 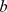 and 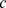 imply large imaginary parts. The spike-based effects on 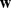 give 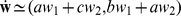. Starting from the homogeneous condition 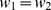, it follows from 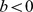 that 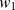 increases faster 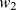. If 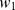 becomes so large that 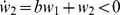, STDP results in LTP for 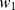 and LTD for 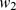 at the end of the learning epoch. This means that, despite an initial growth in the case 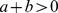 (which is predicted by the eigenvectors), 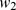 is eventually depressed. In the general case, we also expect that some weights may become depressed because others experience stronger LTP due to STDP. In any case, the most strongly potentiated weights at the initial splitting should eventually be the winners of the synaptic competition.

### Response to correlated inputs after learning

Here we examine the spike transmission after learning, which is used to quantify mutual information in Results. To fix ideas, we present simple calculations for a neuron excited by a correlated pool of 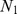 inputs with homogeneous weight 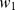 and correlation strength 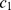. The firing probability during a given time interval of 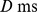 consecutive to a spike from input 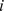, similar to a peristimulus time histogram (PSTH), can be seen as a measure of spike-based information transmission. It amounts to 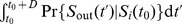, which relates to the correlation term of 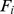, namely 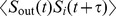, evaluated for 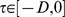 and rescaled by the spike rate of 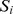. For an isolated spike at time 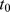, i.e., outside a correlated event such as that related to a reference 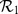 in (11), the above integral can be approximated by
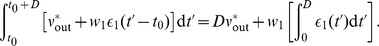
(47)Likewise, for a spike involved in a correlated event, the average increase of firing probability is scaled up by the mean number of coincidentally firing inputs:
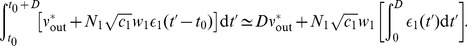
(48)When the neuron has many inputs and a non-zero background firing activity, the group effect dominates with 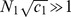, so we can neglect the term in 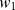 in (48). The ratio between (49) and (48) then becomes
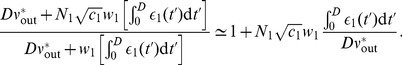
(49)To maximize this ratio, the optimal 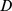 lies beneath the values for which most of the integral of 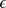 is covered. Larger values for 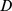 beyond the timescale of the PSP kernel (e.g., several hundreds of ms as used for rate-based coding) lead to a smaller gain. With our parameters, we choose 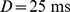 such that 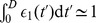. The lower the equilibrium mean firing rate 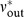, the stronger this signal-to-noise ratio is. For the Poisson neuron, 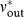 is also the variance of the firing rate, which can also be thought as a source of noise for rate coding. Note that from (26) with plain STDP, the equilibrium weight 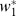 for a pool of 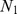 instantaneously correlated inputs with strength 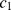 satisfies 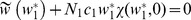, which gives a theoretical prediction of the expressions above.

### Supporting information

**Text S1.** This section focuses on the situation where the spectrum of 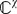 contains imaginary eigenvalues. For add-STDP, this can lead to an oscillatory-like behavior of the weights. In contrast, weight-dependent STDP stabilizes the weight distribution.

**Figure S1.** Example of quasi-periodic evolution for plastic weights modified by add-STDP.

**Text S2.** We show that spike coordination and rate covariation can induce correlations of similar strength. We consider a neuron stimulated by inputs that have a common periodic firing rate. We show how the weight evolution is determined by the frequency of the input rate, the postsynaptic response and the STDP learning window.

**Figure S2.** Example of weight evolution that depends on the frequency of oscillatory input firing rates. The postsynpatic neuron can be trained to represent only a certain frequency range, similar to a band-pass filter.

## Supporting Information

Figure S1Instability of the emerging weight distribution. (A) The postsynaptic neuron is stimulated by 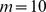 pools. Nine pools exhibit correlated activity such that their respective inputs tend to fire in sequence a common reference 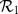, namely after a time lag equal to 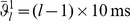 for pool 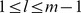; cf. (11) in the main text. The tenth pool has no correlation (x). (B) Spectrum of the corresponding input covariance matrix 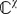. Comparison of the weight evolution for (C) add-STDP+SCC and (D) nlta-STDP+SCC with 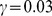. The other STDP parameters are the same for both models: 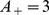; 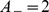; 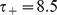 and 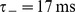; 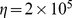; 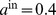; 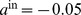; 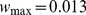. (1) The weight traces are represented in light gray. The mean weights over each input pool are in darker gray, apart from three that are displayed in color (thicker line). (2) Normalized distribution of the mean weights over each pool at different time of the simulation (time is indicated in thousands of seconds on the y-axis; blue corresponds to the earliest). For C1, the blue curve for 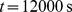 has been reproduced in dotted line to compare it with distributions at two later times.(EPS)

Figure S2Competition between instantaneous correlations and oscillatory firing rate. (A) Schematic representation of a single neuron stimulated by pool 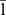 with instantaneous correlations and pool 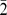 with an oscillatory firing rate (frequency 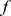). Their respective cross-correlograms are represented in red and blue. (B) Plot of the correlogram 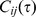 between two oscillatory inputs from pool 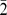 (blue trace). The two spike trains were simulate for 1000 s and the time bin for the x-axis is 1 ms. The predicted curve (black dashed line) corresponds to (S2) in Text S2: a cosine function with frequency 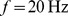 and amplitude 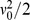 with 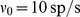. (C) Theoretical prediction of the mean input-neuron correlation coefficients 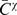 for each input pool. The blue solid curve corresponds to the oscillatory pool 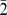 and varies with the frequency 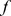, cf. (S3) in Text S2. The red dashed and dashed-dotted horizontal lines represent the instantaneous correlation of 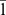 for 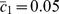 and 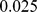, respectively; cf. (13) in the main text. The input firing rate is 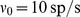 for both pools. (D) Plots of the difference between the mean weights after 500 s of simulated time. Purple circles and green crosses correspond to 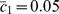 (dashed line in B) and 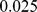 (dashed-dotted line in B), respectively. Positive values indicate that the oscillatory pool is the winner at the end of the learning epoch. (E) Effect of dendritic delays on the STDP effect. The solid line is the same as in the case with 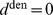, whereas the dashed and dashed-dotted curves correspond to 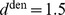 and 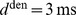, respectively.(EPS)

Text S1This section focuses on the situation where the spectrum of 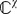 contains imaginary eigenvalues. For add-STDP, this can lead to an oscillatory-like behavior of the weights. In contrast, weight-dependent STDP stabilizes the weight distribution.(PDF)

Text S2 We show that spike coordination and rate covariation can induce correlations of similar strength. We consider a neuron stimulated by inputs that have a common periodic firing rate. We show how the weight evolution is determined by the frequency of the input rate, the postsynaptic response and the STDP learning window.
(PDF)
