# Supplementary material for: Spectral Analysis of Input Spike Trains by Spike-Timing-Dependent Plasticity
Source: PLoS Comput Biol. 2012 Jul 5;8(7):e1002584. doi: 10.1371/journal.pcbi.1002584 (PMC3390410; doi:10.1371/journal.pcbi.1002584)
Supplement: Text S1 — This section focuses on the situation where the spectrum of contains imaginary eigenvalues. For add-STDP, this can lead to an oscillatory-like behavior of the weights. In contrast, weight-dependent STDP stabilizes the weight distribution. (PDF) [file pcbi.1002584.s003.pdf]

Spectral Analysis of Input Spike Trains by Spike-Timing-Dependent Plasticity: Supplemental Information S1

Matthieu Gilson[1,2,*], Tomoki Fukai[1], Anthony N Burkitt[2,3,4]
1 Riken Brain Science Institute, Wako-shi, Saitama 351-0198, Japan
2 NeuroEngineering Laboratory, Department of Electrical and Electronic Engineering, The University of Melbourne, Melbourne, VIC 3010, Australia
3 Centre for Neural Engineering, The University of Melbourne, Melbourne, VIC 3010, Australia
4 Bionics Institute, 384-388 Albert Road, East Melbourne, VIC 3002, Australia
∗ E-mail: gilson@brain.riken.jp; gilsonm@unimelb.edu.au

Oscillatory weight dynamics due to imaginary eigenvalues in the spectrum

This section focuses on the situation where the spectrum of $\mathbb{C}^\chi$ contains imaginary eigenvalues. For add-STDP+SCC, the theoretical analysis showed a possible instability in the synaptic dynamics. To examine this issue, we consider the configuration in Fig S1A, for which inputs fire in sequence, leading to many eigenvalues with large imaginary parts (Fig S1B). In the simulation in Fig S1C1, the weight means for pools $\bar{3}$ to $\bar{9}$ experience large fluctuations, as illustrated by the thick colored curves for three pools. The distribution of means exhibits an oscillatory-like behavior: the distribution of means in Fig S1C2 are almost identical at times $t = 12000$ and 37000 s (solid red and dotted blue curves), whereas the distribution of weight is in "antiphase" at $t = 27000$ s for pools $\bar{5}$ to $\bar{9}$ (solid purple). (Note that the observed oscillations are sensitive to, e.g., the upper bound enforced on the weights here. Because of the positive real part of the eigenvalues in Fig S1B, the amplitude of the weight oscillations may increase and the weights may get stuck at either bound.) However, nlta-STDP+SCC with very weak weight dependence ($\gamma = 0.03$ instead of 0) generates a rather stable weight structure (Fig S1D), despite the noise in the synaptic dynamics. Even a slight dose of weight dependence in STDP provides stability in the synaptic dynamics, as was predicted by our theoretical analysis of the fixed point.

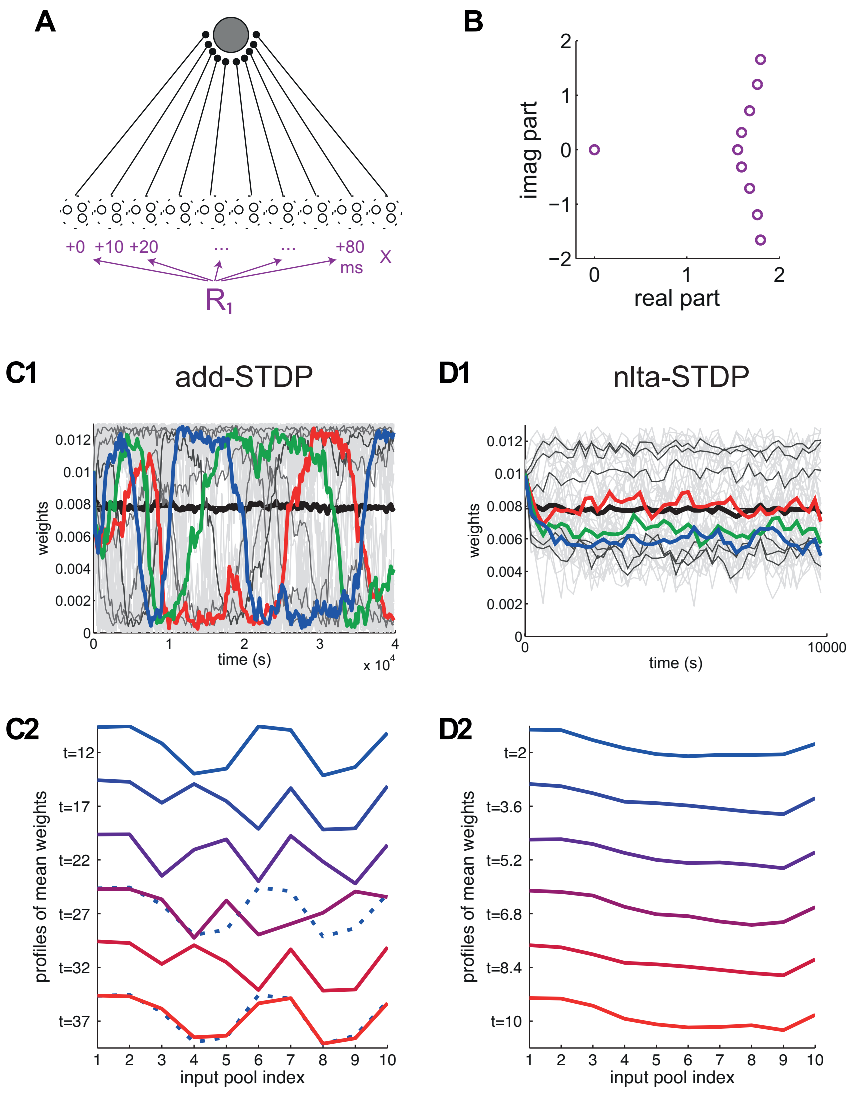

Figure S1. Instability of the emerging weight distribution. (A) The postsynaptic neuron is stimulated by $m = 10$ pools. Nine pools exhibit correlated activity such that their respective inputs tend to fire in sequence a common reference $\mathcal{R}_1$, namely after a time lag equal to $\bar{\vartheta}_l^1 = (l-1) \times 10$ ms for pool $1 \leq l \leq m-1$; cf. (11) in the main text. The tenth pool has no correlation (x). (B) Spectrum of the corresponding input covariance matrix $\mathbb{C}^\chi$. Comparison of the weight evolution for (C) add-STDP+SCC and (D) nlta-STDP+SCC with $\gamma = 0.03$. The other STDP parameters are the same for both models: $A_+ = 3$; $A_- = 2$; $\tau_+ = 8.5$ and $\tau_- = 17$ ms; $\eta = 2 \times 10^5$; $a^{\text{in}} = 0.4$; $a^{\text{in}} = -0.05$; $w_{\max} = 0.013$. (1) The weight traces are represented in light gray. The mean weights over each input pool are in darker gray, apart from three that are displayed in color (thicker line). (2) Normalized distribution of the mean weights over each pool at different time of the simulation (time is indicated in thousands of seconds on the y-axis; blue corresponds to the earliest). For C1, the blue curve for $t = 12000$ s has been reproduced in dotted line to compare it with distributions at two later times.
