# Supplementary material for: Spectral Analysis of Input Spike Trains by Spike-Timing-Dependent Plasticity
Source: PLoS Comput Biol. 2012 Jul 5;8(7):e1002584. doi: 10.1371/journal.pcbi.1002584 (PMC3390410; doi:10.1371/journal.pcbi.1002584)
Supplement: Text S2 — We show that spike coordination and rate covariation can induce correlations of similar strength. We consider a neuron stimulated by inputs that have a common periodic firing rate. We show how the weight evolution is determined by the frequency of the input rate, the postsynaptic response and the STDP learning window. (PDF) [file pcbi.1002584.s004.pdf]

Spectral Analysis of Input Spike Trains by Spike-Timing-Dependent Plasticity: Supplemental Information S2

Matthieu Gilson[1,2,*], Tomoki Fukai[1], Anthony N Burkitt[2,3,4]
1 Riken Brain Science Institute, Wako-shi, Saitama 351-0198, Japan
2 NeuroEngineering Laboratory, Department of Electrical and Electronic Engineering, The University of Melbourne, Melbourne, VIC 3010, Australia
3 Centre for Neural Engineering, The University of Melbourne, Melbourne, VIC 3010, Australia
4 Bionics Institute, 384-388 Albert Road, East Melbourne, VIC 3002, Australia
∗ E-mail: gilson@brain.riken.jp; gilsonm@unimelb.edu.au

Oscillatory input spike trains versus instantaneous correlation

Here we compare the correlation strengths of spike coordination (used in the main text) and periodic rate covariation. We focus on spiking activity that simply arises from oscillatory instantaneous firing rates that have the same frequency. We show how spike-time correlations between such spike trains contain information about their common frequency, which affects the STDP dynamics.

We consider the situation where the postsynaptic neuron in Fig S2A receives input from two pools, pool $\bar{1}$ having instantaneous correlations and pool $\bar{2}$ exhibiting an oscillatory population firing rate. The input spike trains $S_i(t)$ from pool $\bar{2}$ are generated by an oscillatory rate function with frequency f, as sketched in Fig S2A:

$$\rho_i(t) \,=\, \nu_0 \left[1 + \cos(2\pi f t)\right] \ . \tag{S1}$$

The corresponding mean firing rate is $\nu_i(t) = 1/T \int_{t-T}^{t} \rho_i(t')\,\mathrm{d}t' \simeq \nu_0 = 10$ sp/s, when the averaging period T is sufficiently large ($fT \gg 1$). Periodic spiking activity induces spike-time correlations between inputs, as shown in in Fig S2B. The covariance function C_{ij} defined for two inputs i and j is given by

$$C_{ij}(t,\tau) \,=\, \frac{1}{T}\int_{t-T}^{t} \nu_0^2 \left[1+\cos(2\pi f t')\right]\left[1+\cos(2\pi f t' + 2\pi f \tau)\right]\,\mathrm{d}t' \,-\, \nu_0^2 \,\simeq\, \frac{\nu_0^2}{2}\cos(2\pi f\tau) \tag{S2}$$

In the expression for $C_{ij}(t,\tau) \simeq C_{ij}(\tau)$, only the product of the cosine functions contribute, carrying information about f. Like the firing rate, the spike-time covariance is almost independent of the time variable t for sufficiently large T. We have used cosine functions for simplicity of calculation, but any quasi-periodic function could be used here. Note that periodic spike trains with distinct non-harmonic frequencies would have a flat correlogram. Phase shifts between oscillatory inputs would also appear in (S2), but this will no be examined here. Detailed epxressions for the firing rates and correlations above are provided Methods; see (34) and (35).

Combining (4) in the main text and (37) in Methods, the corresponding coefficient for inputs i and j belonging to the oscillatory pool $\bar{2}$ is a cosine transform of the kernel χ assumed to be identical for all synapses:

$$C_{ij}^{\chi} \,\simeq\, \int_{-\infty}^{+\infty} \chi\left(\tau - 2d^{\mathrm{den}}\right)\,\frac{\nu_0^2}{2}\cos(2\pi f\tau)\,\mathrm{d}\tau \,=\, \frac{\nu_0^2}{2}\,\Re\left\{\hat{\chi}(f)\exp\left(4\pi\mathrm{i}d^{\mathrm{den}}f\right)\right\} \ , \tag{S3}$$

where $\Re$ denotes the real part of complex numbers, $\mathrm{i} = \sqrt{-1}$ and the hat denotes the Fourier transform $\hat{g}(f) = \int_{-\infty}^{+\infty} g(\tau)\exp(-2\mathrm{i}\pi\tau f)\,\mathrm{d}\tau$.

To predict the weight evolution, we compare the covariance $\bar{\mathbb{C}}^\chi$ for each pool, as illustrated in Fig S2C. We adjust the correlation strength of pool $\bar{1}$ such that the STDP effects are comparable for both pools. The dashed and dashed-dotted horizontal lines correspond to $\bar{c}_1 = 0.05$ and 0.025, respectively. Because the STDP effect evaluated in (S3) depends on the input frequency f, so does the synaptic competition between the two pools. Note that high frequencies outside the range of STDP, $f \geq 100$ Hz here, lead to very weak contributions. The difference between the mean weights from pools 2 and 1 is plotted in Fig S2D after 500 s of simulation. Positive values indicate that the oscillatory pool wins. The simulation results agree with the prediction that the oscillatory pool wins when the solid curve is above the horizontal line corresponding to $\bar{c}_1$. Circles and crosses in Fig S2D correspond to the dashed and dashed-dotted lines in Fig S2C, respectively. As a result, the frequency band for which the neuron is sensitive can be adjusted via $\bar{c}_1$. Note that we use add-STDP+SCC as the correlation strengths are rather weak here.

The dendritic delay d^{den} shifts both the learning window W and the PSP response kernel ϵ, such that its lumped effect for feedforward connections is twice the shift, cf. (4) in the main text. Recall that, in contrast, the effects of axonal delays eventually cancel out. The curves of (S3) for various delays and time constants are plotted in Fig S2E. The frequency dependence of the STDP specialization can be used to build a neuronal frequency selector. An in-depth study of the weight dynamics for inhibitory STDP can be found in Gilson et al. [1] where both heterogeneous delays and PSP time constants are used.

References

1. Gilson M, Bürck M, Burkitt AN, van Hemmen JL (2012) Frequency selectivity emerging from spike-timing-dependent plasticity. Neural Computation (in press).

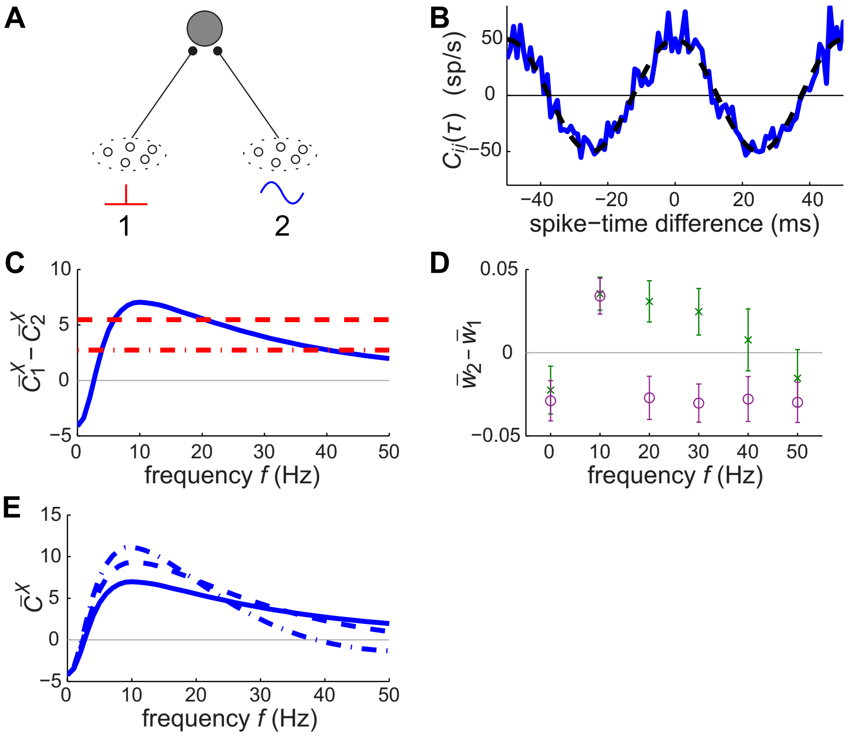

Figure S2. Competition between instantaneous correlations and oscillatory firing rate. (A) Schematic representation of a single neuron stimulated by pool $\bar{1}$ with instantaneous correlations and pool $\bar{2}$ with an oscillatory firing rate (frequency f). Their respective cross-correlograms are represented in red and blue. (B) Plot of the correlogram $C_{ij}(\tau)$ between two oscillatory inputs from pool $\bar{2}$ (blue trace). The two spike trains were simulate for 1000 s and the time bin for the x-axis is 1 ms. The predicted curve (black dashed line) corresponds to (S2): a cosine function with frequency $f = 20$ Hz and amplitude $\nu_0^2/2$ with $\nu_0 = 10$ sp/s. (C) Theoretical prediction of the mean input-neuron correlation coefficients $\bar{C}^X$ for each input pool. The blue solid curve corresponds to the oscillatory pool $\bar{2}$ and varies with the frequency f, cf. (S3). The red dashed and dashed-dotted horizontal lines represent the instantaneous correlation of $\bar{1}$ for $\bar{c}_1 = 0.05$ and 0.025, respectively; cf. (**??**). The input firing rate is $\nu_0 = 10$ sp/s for both pools. (D) Plots of the difference between the mean weights after 500 s of simulated time. Purple circles and green crosses correspond to $\bar{c}_1 = 0.05$ (dashed line in B) and 0.025 (dashed-dotted line in B), respectively. Positive values indicate that the oscillatory pool is the winner at the end of the learning epoch. (E) Effect of dendritic delays on the STDP effect. The solid line is the same as in the case with $d^{\mathrm{den}} = 0$, whereas the dashed and dashed-dotted curves correspond to $d^{\mathrm{den}} = 1.5$ and $d^{\mathrm{den}} = 3$ ms, respectively.
